# KDM7A and KDM1A inhibition suppresses tumour promoting pathways in prostate cancer

**DOI:** 10.1002/1878-0261.70238

**Published:** 2026-03-23

**Authors:** Jennie N Jeyapalan, Veronika M Metzler, Simone de Brot, Corinne L Woodcock, Anna E Harris, Jennifer Lothion‐Roy, Emeli M Nilsson, Atara Ntekim, Michael S Toss, Jenny L Persson, Francesca Khani, Brian D Robinson, Lorraine J Gudas, Emad Rakha, David M Heery, Catrin S Rutland, Nigel P Mongan

**Affiliations:** ^1^ School of Veterinary Medicine and Sciences, University of Nottingham Sutton Bonington UK; ^2^ Biodiscovery Institute, University of Nottingham UK; ^3^ COMPATH, Institute of Animal Pathology, University of Bern Switzerland; ^4^ Department of Oncology University Hospital Ibadan Nigeria; ^5^ Department of Molecular Biology Umeå University Sweden; ^6^ Department of Biomedical Sciences Malmö Universitet Sweden; ^7^ Department of Urology Weill Cornell Medicine New York USA; ^8^ Department of Pharmacology Weill Cornell Medicine New York USA; ^9^ School of Pharmacy, University of Nottingham UK

**Keywords:** androgen receptor, castration resistant prostate cancer (CRPC), co‐regulation, epigenetics, Lysine demethylases, therapeutics

## Abstract

Treatment resistance has become a major challenge in cancer research, particularly for patients with advanced castration resistant prostate cancer (CRPC) where no curative therapies are available. Epigenetic alterations play a significant role in cancer progression. In prostate cancer (PCa), where androgen receptor (AR) is the primary oncogenic driver, epigenetic coregulators, specifically lysine demethylases (KDMs), have previously been identified as factors that alter the transcriptome as cancer cells acquire resistance. KDM7A has been identified as a cancer‐promoting factor in many cancers; however, its role in PCa remains largely unexplored. This study investigates the clinical relevance of KDM7A in comparison with the well‐studied KDM1A in PCa. Using PCa cell line models, we confirm KDM7A as an AR coregulator. By exploiting commercially available pharmacological inhibitors, we demonstrate that in AR‐positive CRPC cell lines, combinatory inhibition of KDM1A and KDM7A leads to a loss of AR and the AR‐driven transcriptome, which in turn attenuates cancer‐promoting cell phenotypes. These findings highlight the potential of combination‐targeted therapies in tackling advanced prostate cancers.

## Graphical abstract

Treatment resistance is a major challenge for patients with advanced prostate cancer. This study examined an alternative approach to target the major prostate cancer‐promoting pathway by targeting epigenetic factors, whose levels are higher in tumours. Utilising cell line models, we were able to show that inhibition of these factors supported tumour suppression, loss of cell growth and metastatic potential.

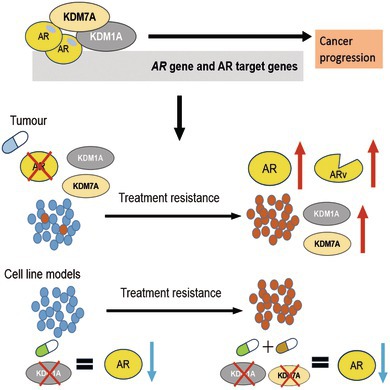

**Abbreviations**
A3SS: alternative 3′ splice site
A5SS: alternative 5′ splice site
AR: androgen receptor
ATM: ataxia telangiectasia mutated
BRCA1: BRCA1 DNA repair associated
CRPC: castration‐resistant prostate cancer
FOXA1: forkhead box A1
H3K27: histone 3 lysine 27
H3K4: Histone 3 lysine 4
H3K9: histone 3 lysine 9
KDM1A: lysine demethylase 1A
KDM2A: lysine demethylase 2A
KDM7A: lysine demethylase 7A
KDM7B: lysine demethylase 7B
KLK3: kallikrein related peptidase 3
MAPK: mitogen‐activated protein kinase
MXE: mutally exclusive exon
NEPC: neuroendocrine prostate cancer
NKX3.1: NK3 homeobox 1
P53: tumour protein 53
PCa: prostate cancer
PHF8: PHD finger protein 8
PSA: prostate‐specific antigen
RAD21: RAD21 cohesin complex component
RI: retained intron
SE: skipped exon
TCGA: the cancer genome atlas
TGF‐β: transforming growth factor Beta
TMPRSS2: transmembrane serine protease 2
VEGF: vascular endothelial growth factor

## 1 Introduction

Prostate cancer (PCa) is driven by androgen and androgen receptor (AR). Current treatments target androgen production, androgen deprivation therapies (ADT) and AR signalling (AR signalling inhibitors, ARSI), with new therapeutic avenues being developed and trialled in the clinic [1, 2]. Currently, no curative treatment options exist for metastatic castration‐resistant prostate cancer (CRPC) with PCa progression and drug resistance remaining a critical issue [3, 4]. Therefore, identifying new drug targets and developing novel therapies is crucial. The androgen receptor is a nuclear receptor that contains an N‐terminal transactivation domain and DNA binding domain, with the ligand binding domain within the C terminus [5]. Upon binding to ligand, androgens, AR forms dimers that are crucial for its transactivation function [6], with recent interest in targeting the dimerisation to prevent AR function [7]. AR also requires binding to several coregulators for modulating transcription of its target genes [8, 9]. Epigenetic coregulators of the androgen receptor (AR) have therefore gained significant interest as potential targets for PCa [10, 11, 12, 13]. One such coregulator is the histone lysine demethylase KDM1A (also known as LSD1), which has been extensively studied in PCa [14, 15, 16, 17, 18, 19, 20, 21]. KDM1A is capable of demethylating mono‐ and dimethylated H3K4 and H3K9 functioning as both a transcriptional corepressor and coactivator [14, 22]. KDM7A (KIAA1718), a member of the KDM7 family, also demethylates H3K9me2 and targets H3K27me2 [23]. The PHD domain of KDM7A binds to H3K4me3, and when present on the same histone peptide, KDM7A preferentially demethylates H3K27me2 over H3K9me2, thereby acting as a co‐activator [24]. KDM7A has also been implicated in various cancers [25, 26, 27], particularly in breast cancer [28, 29, 30]. However, little is known about the role of KDM7A in prostate cancer, but Lee *et al*. showed that KDM7A acts as an AR coregulator at AR target genes in an androgen dependent PCa cell line, LNCaP, and was present at higher levels in 70 PCa specimens [31].

Our study builds on these findings by examining KDM7A in two PCa cohorts (*n* = 104 and 396 tumours). We investigated the role of KDM7A in PCa in comparison with the well‐studied AR coregulator, KDM1A and further demonstrated that targeting KDM7A and KDM1A in a combination using pharmacological inhibitors deregulated the AR targeted transcriptome, leading to reduced proliferation and invasion. These preclinical findings provide evidence for the therapeutic potential of targeting lysine demethylases in combination for CRPC.

## 2 Materials and methods

### 2.1 Ethics statement and patient tissue specimens

Ethical permissions for the study were granted under the School of Veterinary Medicine and Sciences, University of Nottingham CARE committee (approval numbers: 3483 211 102; 1533 150 901; 1861 161 006) and the Weill Cornell Medicine Institutional Review Board (approval: 1008011210). Both cohorts had written consent of each subject and understanding of the studies that will be undertaken. Nottingham tissue microarray (TMA; cohort 1) was produced from PCa specimens from the Nottingham University Hospital biobank at NHS trust (approval#: ACP0000184; specimens from 2003 to 2007). Cohort 1 consisted of nonmalignant prostate (*n* = 56) and PCa (*n* = 104) specimens. Demographics are provided in Table 1A. The Weill Cornell PCa TMA cohort (cohort 2) comprised African American and Caucasian non‐malignant (*n* = 154) and primary PCa (*n* = 396) specimens, with demographics and clinical characteristics available for 171 patients (Table 1B). Cohort 2 samples were collected from Weill Cornell Medicine prior to 2021 under IRB (approval 1 008 011 210). The Helsinki Declaration of Human Rights and the UK Human Tissue Act were strictly observed.

**Table 1:** PCa patient demographics where clinical parameters are known for (A) Nottingham, UK cohort and (B) Weill Cornell medicine cohort, NY, USA.

Clinicopathological parameters	Frequency *N* (%)
(A) Nottingham PCa TMA	
Age	
< 60	44 (42.3%)
≥ 60	60 (57.7%)
Race	
White	94 (95.9%)
Mixed/Black Caribbean/other	4 (4.1%)
Gleason score	
6	12 (11.7%)
≥ 7	91 (88.3%)
pTNM	
T1 and T2	66 (66.7%)
T3	33 (33.3%)
Biochemical recurrence	
No	59 (64.1%)
Yes	33 (35.9%)
High‐grade PIN	
No	32 (30.7%)
Yes	72 (69.2%)
(B) WCM PCa TMA	
Age	
< 60	69 (40%)
≥ 60	102 (60%)
Race	
White	69 (40%)
African American	102 (60%)
Gleason	
6	29 (17%)
≥ 7	142 (83%)
pTNM	
T2	129 (75%)
T3	42 (25%)
BCR	
No	123 (72%)
Yes	24 (14%)
Not known	24 (14%)

### 2.2 CBioportal analysis of publicly available PCa datasets

The cBioportal webtool [32], was used to examine genetic alterations in *KDM7A* and *KDM1A* in the three PCa cohorts; (1) TCGA prostate adenocarcinomas cohort (Firehose legacy, *n* = 491 patients); (2) Metastatic prostate adenocarcinomas (SU2C/PCF Dream team, *n* = 429 patients) [33]; (3) Neuroendocrine PCa (Multi‐Institute cohort, *n* = 81 patients) [34]. Genetic alterations (copy number variation, mutations) and mRNA levels, where available, were assessed (cBioportal accessed 25/11/2024). Utilising GISTIC, capped linear copy number value graphs, with correlation coefficient analysis (Spearman and Pearson correlations) were used to assess correlation between copy number variants (CNV) and mRNA levels of *KDM7A* and *KDM1A*.

### 2.3 Immunohistochemistry of PCa patient specimens

The immunohistochemical staining was performed using the Novolink™ Max Polymer Detection System (#RE7280‐K; Leica Biosystems, UK) on the TMA slides for both cohorts, with KDM7A primary antibody (#NBP1‐81382, 1 : 100 dilution; Novus biologicals, Canada) and KDM1A (#NB100‐1762, 1 : 1000 dilution; Novus biologicals, Canada) incubated for 1 h at room temperature. High‐resolution scans were taken. The antibodies were chosen due to having been previously published, KDM7A primary antibody (#NBP1‐18382) [26, 35] and KDM1A primary antibody (#NB100‐1762) [16, 36, 37]. Following Optimisation on optimisation slides containing prostate malignant and nonmalignant tissue alongside tongue, liver, appendix and kidney tissues, the stained TMAs were assessed using *H*‐score ([percentage cell positivity 0–100% × intensity 0–3], no staining to high intensity respectively, producing *H*‐scores between 0 and 300). Independent assessment of 10% of cores was additionally undertaken to investigate interobserver variability (analysed using Cronbach's alpha in SPSS and Spearman's rank‐order correlation). Clinical relevance of the KDM7A and KDM1A levels in the patient specimens was determined using *χ*2‐tests (asymptotic significance, 2‐sided) using IBM^®^ SPSS^®^ Statistics, Version 24 (IBM, USA).

We also assessed KDM7A in hydrogel embedded formalin‐fixed CRPC and NEPC cell pellets to validate antibody. Protocol involved cell culture of PCa cell lines as shown in Section 2.4. The cells from four 150 cm dishes were harvested using 5 mm EDTA; the cell pellet (~ 10 million cells) was washed in 1× PBS before pelleting using centrifugation. The cells were fixed using 10% formalin‐neutral buffer for 24 h and then pelleted using centrifugation. The hydrogel (Histogel #HG‐4000‐012; Epredia, UK) was warmed to 60 °C to dissolve the gel and cooled to 50 °C before adding to the cells in a 1 : 4 ratio (cells to hydrogel). The hydrogel cell pellets containing C4‐2, LASCPC‐01, VCaP and 22RV1 were postfixed in 10% formalin, dehydrated through ethanol series, embedded into paraffin blocks and sectioned at 7 μm. IHC was performed using KDM7A antibody (#NBP1‐81382, 1 : 100 dilution) with the Leica Novolink polymer detection kit (Leica, Germany) according to manufacturer's instructions. Antigen retrieval was achieved using sodium citrate buffer. Separate sections underwent immunohistochemistry with no counterstain. Microscopy was carried out to confirm positive cytoplasmic and/or nuclear staining (Leica, Germany). Negative controls for each cell line received no primary antibody and were incubated in fetal bovine serum only. Figure S1A–C shows the IHC KDM7A results for 22RV1, LASCPC‐01, and VCaP, with negative controls shown (Fig. S1D–F respectively). The western blot results for the extracted protein levels from the same pool of cells harvested for the IHC are shown in Fig. S1G (method Section 2.8).

### 2.4 Immunofluorescence validation of KDM7A IHC antibody

Immunofluorescence was performed to validate the KDM7A IHC antibody (#NBP1‐81382) as detailed in Section 2.3. C4‐2 cell line was grown in complete medium as stated in Section 2.5. C4‐2 were plated onto glass coverslips and allowed to adhere for 12 h. Knockdown was performed as stated in Section 2.6 using 25 nm of ON‐TARGETplus siRNA SMART pool assays (Horizon Discovery, UK), targeting *KDM7A* (#L‐025357‐01‐0005) and nontargeting siSCR control (#D001810‐10‐05) plus Dharmafect 2 transfection reagent for 72 h. After 72 h, fixation and permeabilization was carried out using ice‐cold methanol. All incubations were for 1 h at room temperature. Cells were washed in 1xPBS, then blocked in 5% normal goat serum, 0.3% Trition X‐100. Primary antibody (0.5 μg·mL^−1^ KDM7A antibody, #NBP1‐81382 1% goat serum) was added, with washes in 1 × PBS. Final incubation was performed with the secondary antibody (1 : 200, Fluorescein‐conjugated goat anti‐rabbit IgG, #SA00003‐2; Proteintech, UK). Coverslips were mounted with DAPI and imaged using the DM5000b microscope (Leica Microsystems, UK), shown in Fig. S1H.

### 2.5 Cell lines, culture conditions and pharmacological inhibitor treatments

Cell lines were obtained as stated; PNT1A (RRID:CVCL_2163) was kindly donated by Dr Jenny Persson (Umeå University), representing nonmalignant epithelial cells for normalisation. LNCaP (RRID:CVCL_0395) was purchased from ECACC (#8911021; ECACC, UK) and 22RV1 (RRID:CVCL_1045) was purchased from ATCC (#CRL‐2505; ATCC, USA). LNCaP:C4‐2 (C4‐2; RRID:CVCL_4782) was kindly donated by Dr. Doug Scherr, Department of Urology, Weill Cornell Medicine. NEPC LASCPC‐01 cell line was purchased from ATCC (#CRL‐3356; ATCC, USA). All cell lines had been authenticated in the past three years utilising the cell authentication services provided by Eurofins Genomics (Germany). The authentication is performed according to ANSI/ATCC standard ASN‐0002, testing 16 DNA markers using the Applied Biosystems™AmpFLSTR™Plus PCR amplication kit system. Markers were compared to databases (Cello and DSMZ) and certification provided. All the cells were tested for mycoplasma and all experiments were performed with mycoplasma‐free cells.

Cell lines were maintained in RPMI‐1640 complete medium (phenol red RPMI‐1640 supplemented with 10% fetal bovine serum, 1% penicillin–streptomycin, 2 mm L‐Glutamine, and 1 mm sodium pyruvate). For experiments with addition of androgen derivative R1881 (Sigma Aldrich, USA), cells were grown in RPMI‐1640 complete medium (as above, with phenol red free RPMI and substitution of FBS with 10% hormone depleted dialysed FBS). R1881 (1 nm) treatments lasted for 72 h (0.1% ethanol for vehicle). Treatment for 72 h was undertaken with KDM1A inhibitor, Namoline (Abcam, United Kingdom) and KDM7A selective inhibitor, TCE‐5002 (Tocris Bioscience, UK) at concentrations 10–100 μm (0.1% DMSO vehicle, all treatments also had final concentrations of 0.1% DMSO).

### 2.6 Small‐interfering RNA knockdown of *KDM7A* and *KDM1A*

Functional depletion of *KDM7A* and *KDM1A* was performed using the ON‐TARGETplus siRNA SMART pool assays targeting *KDM7A* (#L‐025357‐01‐0005), *KDM1A* (#L‐009223‐00‐0005) and nontargeting control (#D001810‐10‐05) in LNCaP, C4‐2 and 22RV1 cells, utilising the DharmaFECT 2 transfection reagent (Horizon Discovery, UK). Cells were transfected following the manufacturer's protocol, with 10 nm final concentration of the siRNA smartpool mix. Cells were plated (six‐well plate) as stated for R1881 treatment, with both treatment and siRNA incubation for 72 h, before harvesting for RNA and protein extraction.

### 2.7 Gene expression analysis

To assess *KDM7A* and *KDM1A* basal level analysis within PCa cell lines and treated cells, the RNA was extracted from 2 to 3 biological replicates containing *n* = 3 replica wells (6 well plate, *n* = 6–9). RNA was extracted using Trizol (Ambion, UK) or RNeasy kits (Qiagen, USA), with cDNA synthesised using Quanta cDNA synthesis kits (Quantabio, USA). For mRNA expression analysis was performed using the following Taqman™ probes (Thermo Fisher Scientific, USA): Housekeeping gene *GAPDH* Hs03929097_g1; *KDM7A* Hs01398501_m1; *KDM1A* Hs01002741; *KLK3* Hs02576345_m1; *TMPRSS2* Hs01122322_m1; *AR* Hs00171172_m1; *FOXA1* Hs04187555_m1; *NKX3.1* Hs00171834_m1 and the LightCycler® 480 master mix (Roche, Switzerland). The qRT‐PCRs were performed in a LightCycler 480 II (Roche, Switzerland) instrument as previously described [10]. Relative quantification was carried out using the 2(−ΔΔC(T)) method. Statistical analysis was performed using Welch's *t*‐test for comparison between cell lines. Analysis for treatment within the same cell line involving multiple comparisons used one‐way ANOVA (corrected for multiple testing using the Bonferroni *post hoc* method). When comparing between two means only *t*‐test was used. Significance difference in fold change was stated as ≥ 2‐fold difference with *P*‐values < 0.05.

### 2.8 Immunoblotting

For western blotting analysis, proteins were extracted from cells using a SDS and glycerol‐based buffer (100 mm Tris–HCl pH 6.8, 4% SDS, 20% glycerol), with proteins stored at −80 °C. The DCTM (detergent compatible) Protein Assay (BIO‐RAD, USA) was used to quantify protein concentrations (using standard curve method). Protein samples (10–20 μg) were diluted with 5× Laemmli loading buffer prior to PAGE analysis, as published previously [38]. The antibodies and dilutions used were mouse monoclonal anti‐GAPDH (ab9484, 1 : 5000; Abcam, UK); mouse monoclonal anti‐actin (MA515739, 1 : 10000; Invitrogen, UK); rabbit polyclonal anti‐KDM7A (STJ110565, 1 : 1000; St John's Lab, UK); mouse monoclonal anti‐KDM1A (NB100‐1762; Novus Biologicals, UK); rabbit polyclonal anti‐AR (sc‐816; 1 : 5000; Santa Cruz, USA) and secondary antibodies, goat anti‐mouse IgG (ab97023, 1 : 10000; Abcam, UK) or goat anti‐rabbit IgG (ab6721, 1 : 10000, Abcam, UK). Signal detection was performed using Amersham TM ECL Prime reagent (GE Healthcare, USA) and image captured using a ChemiDoc TM MP Imaging System (BIO‐RAD, USA). Quantification of signal intensity used Image studio Lite software (Licor, USA), with mean values across the replicas shown.

### 2.9 ChIP analysis of publicly available datasets

Two studies that undertook ChIP analysis utilising AR antibody were utilised to investigate AR binding and ARE motif identification in PCa cell lines for the *KDM7A* gene. The GEO samples used were GSM698587 (LNCaP), GSM698594 (22RV1), and GSM698598 (VCaP) from GEO series GSE28219 [39] and sample GSM353644 (LNCaP + R1881) from GEO series GSE14097 [40], as confirmation of findings. The sample data was viewed on IGV [41], using genome hg18 (as in the studies).

### 2.10 Phenotypic cell assays for proliferation and invasion

To examine the phenotypic changes caused by inhibitor treatment, the effect on cell proliferation was analysed using the Cyquant NF assay (Invitrogen, UK). The cells were treated for 72 h as stated in Section 2.4 before adding the Cyquant NF dye following the manufacturer's protocol. DNA content relative to vehicle was calculated. To assess the invasive potential of the cells, an *in vitro* transwell‐based assay was utilised. Cell culture inserts (Corning) were coated with Matrigel. Cells were seeded within the transwell inserts with inhibitor and FBS‐free medium; the well contained RPMI‐complete medium plus 10% FBS. The cells were left for 24 h before inserts were methanol fixed and stained with crystal violet, then imaged on an inverted microscope (Leica, UK). Numbers of cells were manually counted from five regions of the insert. Relative invasion was calculated from the vehicle treated cells. A paired *t*‐test was performed.

### 2.11 RNA seq analysis

RNA Seq analysis was performed in biological replicates (*n* = 2) of PCa cells treated with (1) vehicle (DMSO, 0.1%) plus R‐1881 (1 nm); (2) R1881 (1 nm) and namoline (50 μm); (3) R1881 (1 nm) and TCE‐5002 (50 μm); (4) R1881 (1 nm), namoline (50 μm) and TCE‐5002 (50 μm) combination treatment, for C4‐2, with combination treatment only in LNCaP. RNA Seq was also performed on siRNA samples (*n* = 3) for siRNA scrambled plus vehicle (DMSO 0.1%); siRNA scrambled plus R1881 (1 nm); siKDM1A and siKDM7A vehicle (DMSO 0.1%) and siKDM1A and siKDM7A plus R1881 (1 nm). RNA library preparation (mRNA enrichment utilising polyA capture), fragmentation, cDNA reverse transcription, adapter ligation and paired end (PE150) sequencing were carried out by Novogene (Cambridge, UK). Fastq files were quality processed, retaining data with phred > 30, then using TrimGalore adapters were trimmed. Alignment was performed to the human Ensembl annotated reference genome (GRCh38) using the STAR aligner. Differential gene expression was obtained using FeatureCounts [42] and EdgeR [43]. For pathway analysis the RNASeq data was filtered according to log_2_FC ≥ 1 and ≥ −1 respectively with FDR corrected‐*P* value < 0.05 and performed using WebGestalt [44]. Differential splicing analysis was performed using rMATs [45]. The data is available from NCBI‐GEO, accession: GSE194281.

## 3 Results

### 3.1 Clinical relevance of KDM7A and KDM1A in patient cohorts

We first examined the clinical relevance of KDM7A and KDM1A at both mRNA and protein levels in patient cohorts. Utilising the cBioportal webtool [32], *KDM7A* and *KDM1A* genetic alterations were assessed through a comparative analysis of PCa patient cohorts including the TCGA prostate adenocarcinoma cohort (TCGA Firehose legacy), metastatic prostate adenocarcinomas (SU2C/PCF Dream team) and Neuroendocrine PCa cohort (includes CRPC and Neuroendocrine tumours, multi‐institute cohort). Interestingly, in both prostate adenocarcinoma cohorts (primary and metastatic), the most frequent genetic alteration for *KDM7A* was amplification and/or high mRNA (primary adenocarcinomas 5.5% and metastatic PCa 6.1%; Fig. S2A,B). For the neuroendocrine cohort, there were no genetic alterations identified for mutations or copy number for the 81 patients (Fig. S2C). Only 35 patients out of the 81 patients (81 patients = 51 CRPC, 30 NEPC) had mRNA information; of these 21 CRPC and 10 neuroendocrine patients with the corresponding tumours had low mRNA (Fig. S2D). The potential of low KDM7A in neuroendocrine PCa needs to be further investigated in a larger NEPC cohort, at both mRNA and protein level.

**Fig. 1:**
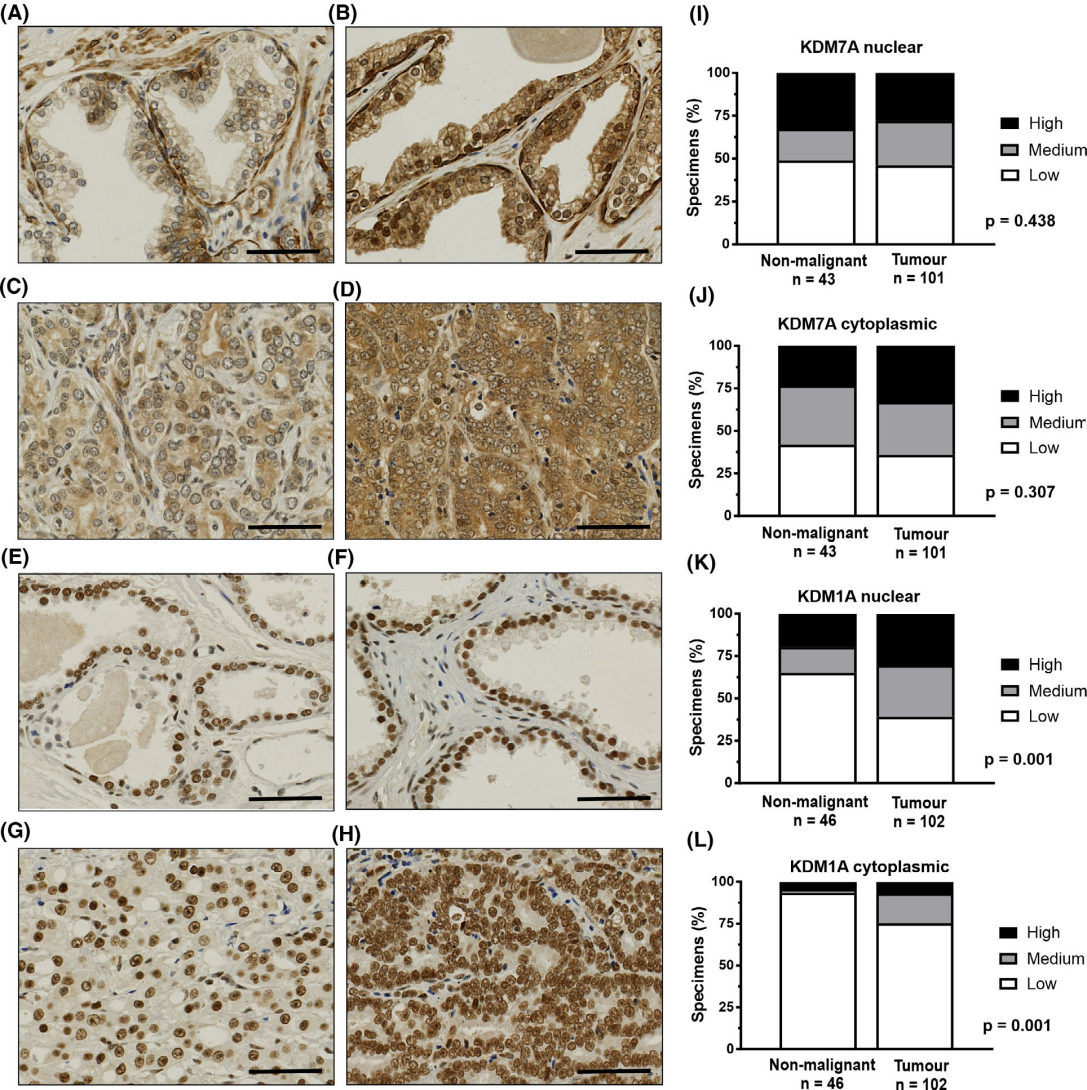
KDM7A and KDM1A levels in UK‐based prostate cancer cohort. To determine the levels of KDM7A and KDM1A in prostate cancer specimens, a tissue microarray (TMA) containing non‐malignant and prostate cancer (Nottingham cohort) was stained for KDM7A and KDM1A. KDM7A and KDM1A staining in non‐malignant and malignant PCa specimens (KDM7A *n* = 43 and *n* = 101; KDM1A n = 46 and *n* = 103 respectively). Representative KDM7A staining non‐malignant tissue (A, B) and tumour tissue (C, D). Representative KDM1A staining non‐malignant tissue (E, F) and tumour tissue (G, H). There was no difference in nuclear (I) and cytoplasmic (J) KDM7A staining between non‐malignant and tumour specimens (Nuclear *H*‐score: low = 25–100, medium = 110–125, high = 130–220; Cytoplasmic *H*‐score: low = 40–100, medium = 110–140, high = 145–270). KDM1A staining was higher (*P* = 0.001) in tumour compared to non‐malignant specimens in both nucleus (K) and cytoplasm (L; Nuclear *H*‐score, low = 90–110, medium = 120–140, high = 150–220; Cytoplasmic *H*‐score, negative = 0, medium = 10–50, high = 90–100). Statistical *P*‐values were determined by *χ*2‐test and log‐rank test. Scale bar = 50 μm.

For *KDM1A*, similar findings to *KDM7A* were observed in primary and metastatic adenocarcinomas, but no alterations were seen in the neuroendocrine cohort (Fig. S2A–C). A comparison between copy number variation and mRNA levels revealed a weak positive correlation for both *KDM7A* and *KDM1A*, with considerable variation within the diploid tumours (Fig. S2E,F). Only three mutations were detected in primary adenocarcinomas for both genes (Fig. S2G,H). These findings suggest that KDM7A overexpression could potentially contribute to tumour growth in a subset of adenocarcinomas but may no longer be necessary or may inhibit progression of advanced tumours. We then examined the protein levels of both KDM7A and KDM1A in two primary adenocarcinoma cohorts (UK and USA). For the analysis we calculated *H*‐scores for each core. The *H*‐score evaluates both the staining intensity and number of cells with positive staining. Initially, *H*‐scores were binned and groupings for low, medium and high staining were then chosen from the staining distribution into evenly matched group numbers where possible (Fig. S3A–D, for KDM7A normal and tumour specimens; Fig. S4A–D for KDM1A normal and tumour specimens respectively). The primary adenocarcinoma cohort 1 showed nuclear and cytoplasmic staining in both nonmalignant and tumour specimens for KDM7A (Fig. 1A–D) and KDM1A (Fig. 1E–H). No significant difference in staining intensity in tumours compared to nonmalignant was exhibited for KDM7A (Fig. 1I,J). KDM1A showed greater staining intensity in the tumour specimens than in non‐malignant tissue (*P* = 0.001; Fig. 1K,L). Assessing clinical characteristics, KDM7A was present at significantly higher levels in tumours with high Gleason scores (Gleason 4 + 3, 8 and 9; Fig. S5A,B). For KDM1A, higher cytoplasmic levels were associated with lower Gleason and extraprostatic extension (Fig. S5C,D). KDM7A and KDM1A levels showed a significant positive correlation (*R* = 0.56, *P* < 0.001; Fig. S5E). We also correlated KDM7A and KDM1A to AR levels; AR *H*‐score results were previously shown in [38]. KDM7A and KDM1A showed a significant positive correlation with AR levels, with greater correlation between KDM1A and AR (KDM7A‐AR, *R* = 0.27, *P* = 0.01; KDM1A‐AR, *R* = 0.55, *P* < 0.001; Fig. S5F,G). We utilised cbioportal to correlate *KDM7A/KDM1A* with *AR* levels in the PCa primary adenocarcinoma TCGA firehose legacy cohort. Interestingly, only *KDM7A*, not *KDM1A*, levels showed a positive correlation with *AR* (Fig. S5H,I).

**Fig. 2:**
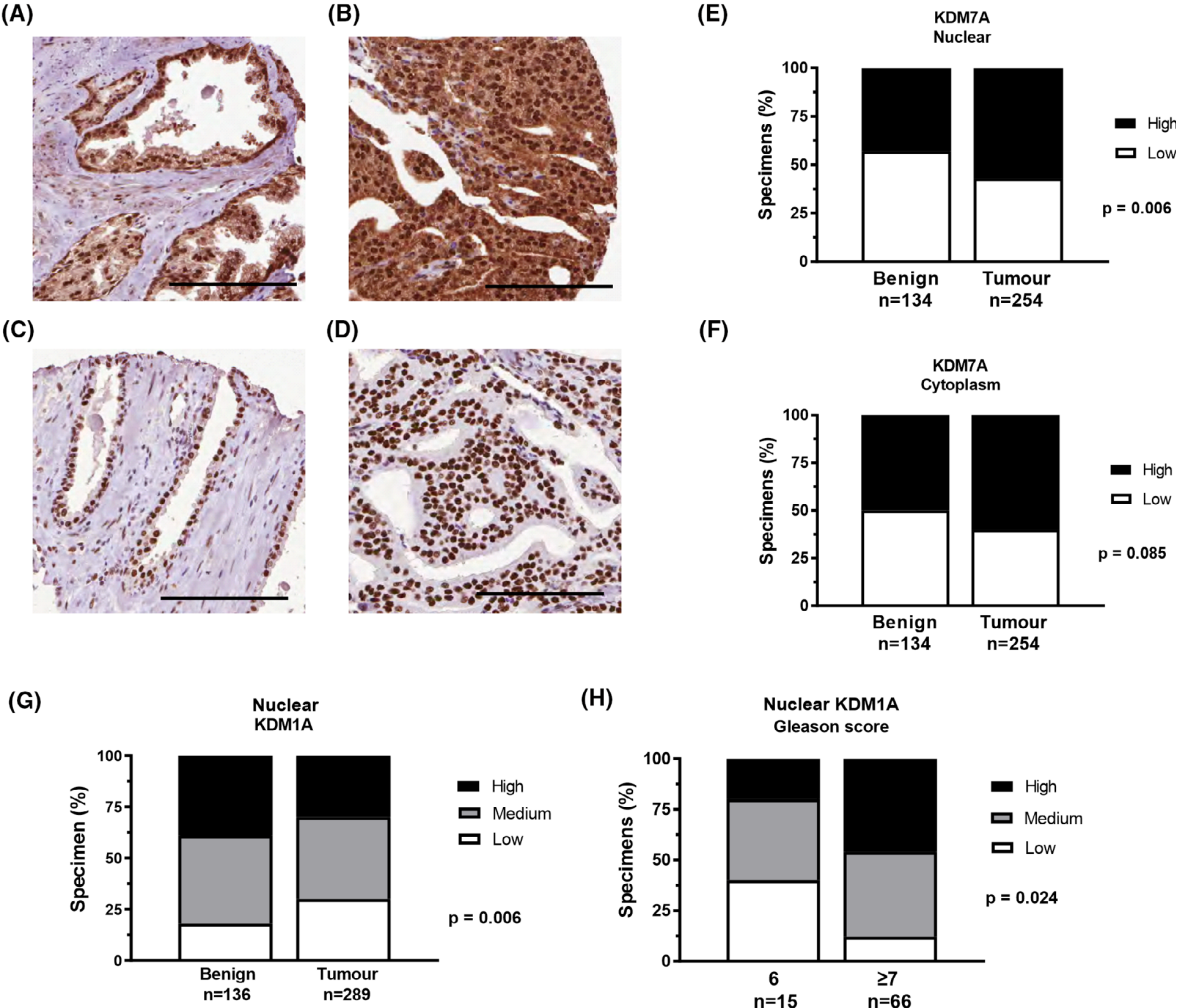
KDM7A and KDM1A levels in African American and Caucasian prostate cancer cohort. KDM7A and KDM1A staining in non‐malignant and malignant PCa specimens (Weill‐Cornell Medicine Cohort, tissue microarray). Representative staining of benign (A, C), tumour tissue (B, D) for KDM7A and KDM1A respectively. There was a significant difference in nuclear (E) but not cytoplasmic KDM7A levels in tumours (F). There were no significant clinical parameters associated with KDM7A. KDM1A only stained within the nucleus, with nuclear levels lower in tumour (G) compared to benign specimens. Within the African American specimens only, KDM1A was significantly higher with gleason grade 7 or above (H). KDM7A *H*‐score: low < 180, high ≥ 190. KDM1A Hscore: low < 180, medium = 190–200, high > 200. Scale bar = 100 μm. Statistical *P*‐values were determined by *χ*2‐test.

In the larger cohort 2, *H*‐score analysis was performed as shown for cohort 1 for KDM7A nuclear and cytoplasmic staining, in non‐malignant and tumour specimens respectively (Fig. 2A,B; Fig. S6A–D). Nuclear staining was only seen for KDM1A within nonmalignant and tumour specimens (Fig. 2C,D; Fig. S6E,F). KDM7A within the nucleus was present at a higher level in tumours than non‐malignant tissue, with no significant clinical correlations identified for specimens where this information was available (Fig. 2E,F). Unlike previous studies, we found that KDM1A levels were lower in tumours than nonmalignant tissue (*P* = 0.006; Fig. 2G). No clinical correlations were identified within the cohort, but analysis of the African American clinical data only showed that KDM1A was significantly higher in tumours with higher Gleason grade (*P* = 0.0024; Fig. 2H). These findings show that KDM7A and KDM1A are present within prostate cancer tumours, that levels are not necessarily higher than in nonmalignant tissue and that there is significant positive correlation in the levels between each other and AR.

### 3.2 KDM7A is androgen regulated and acts as an AR co‐regulator in PCa cell models

**Fig. 3:**
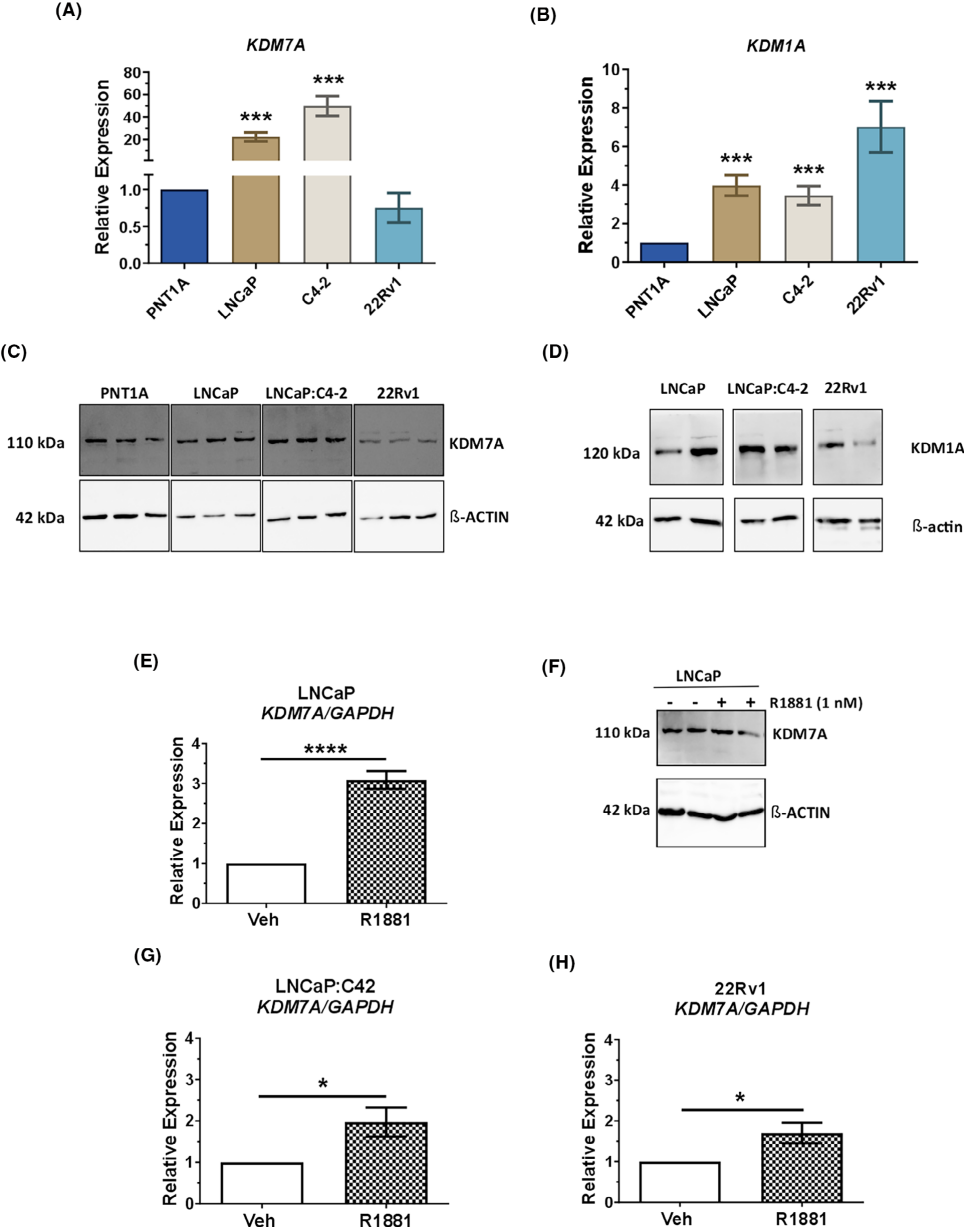
*KDM7A* is regulated by androgen in AR‐positive PCa cell lines. *KDM7A* (A) and *KDM1A* (B) mRNA levels in AR‐positive, androgen‐dependent (LNCaP), androgen‐independent (LNCP:C4‐2 and 22RV1) cell lines compared to PNT1A (normal epithelial SV‐40 immortalised cells). *N* = 9 biological replicas, analysis by unpaired *t*‐test (*t*‐test = *t*). Normalised using *GAPDH*. KDM7A and KDM1A protein was present in the PCa cell lines (C and D respectively) with *β*‐actin as loading control. *KDM7A* expression was up‐regulated upon androgen‐derivative, RI881 treatment in the PCa cell lines, LNCaP (E) but not at the protein level (F). CRPC cell line C4‐2 (G) and 22RV1 (H) also saw up‐regulation of *KDM7A* upon R1881 treatment; *n* = 6 biological replicas *P ≤ 0.05; ***P ≤ 0.001; ****P ≤ 0.0001 by paired *t*‐test. All error bars show SEM.

To further understand the role of KDM7A in prostate cancer progression, we examined the mRNA and protein levels of KDM7A in comparison with KDM1A in prostate cancer cell lines, representing immortalised epithelial cells (PNT1A), AR‐positive, androgen‐dependent PCa (LNCaP), AR‐positive, androgen‐independent CRPC (LNCaP‐derived, C4‐2) and AR‐positive, AR variant positive, androgen‐independent CRPC (22RV1) cells. *KDM7A* was present at higher levels in LNCaP and C4‐2 cell lines compared to PNT1A and 22RV1 (Fig. 3A), whereas *KDM1A* was expressed at significantly higher levels in all the PCa cell lines compared with PNT1A (Fig. 3B). We confirmed the presence of KDM7A and KDM1A protein levels in the PCa cell lines (Fig. 3C,D respectively; full blots for Fig. 3D shown in Fig. S7A). We also examined whether *KDM7A* was regulated by androgen, with mRNA levels increasing after 72‐h treatment in LNCaP cells with androgen derivative R1881 (1 nm final concentration; Fig. 3E) but this was not reflected at protein level (Fig. 3F). For R1881 treated C4‐2 and 22RV1 cells, a subtle significant increase in *KDM7A* was observed (Fig. 3G,H). To examine this further, we utilised published AR ChIP data in PCa cell lines from GSE28219 [39] and GSE14097 [40], to identify whether AR bound *KDM7A*. AR bound within the first intron of *KDM7A* gene (Fig. S7B) and analysis of ARE motifs within this region identified a selective ARE motif within this intron ([46]; Fig. S7C). We next examined KDM7A nuclear and cytoplasmic levels in the cell lines, as KDM7A was present in both locations within PCa specimens. We identified KDM7A presence in both the nuclear and cytoplasmic fractions, but predominately in the cytoplasm (Fig. S7D).

**Fig. 4:**
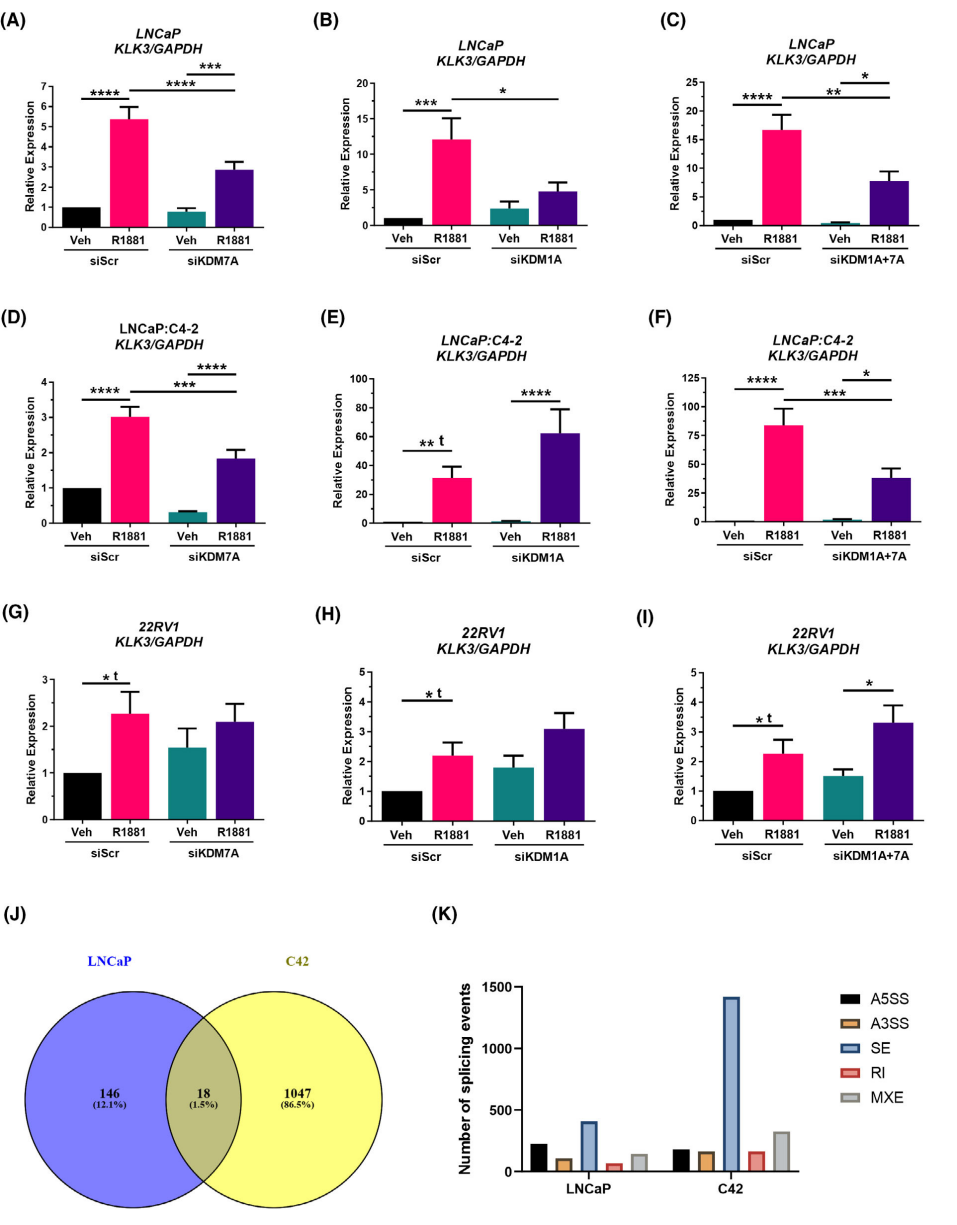
*KDM7A* knockdown shows AR co‐activator function, with combination knockdown of *KDM1A* leading to transcriptomic and alternative splicing. QRT‐PCR measured *KLK3/PSA* levels upon siRNA knockdown of *KDM7A*, *KDM1A* and dual knowdown for LNCaP (A–C), LNCaP:C4‐2 (D–F), and 22RV1 (G–I) respectively. Genes differentially expressed (> 2fold FDR <0.05), in combination knockdown compared between two cell lines LNCaP and LNCaP:C4‐2, Venn diagram (J). (K) Alternative spliced events identified between siScrambled and siKDM7A_siKDM1A. for each cell line. Events are alternative 5′ splice site (A5SS), alternative 3′ splice site (A3SS), skipped exon (SE), retained intron (RI), mutually exclusive exon (MXE). QRT‐PCR *N* = 6 (biological replicas), *P ≤ 0.05; **P ≤ 0.005; ***P ≤ 0.001; ****P ≤ 0.0001 by ANOVA and paired *t*‐test (*t*‐tests are denotated by *t*) with all error bars showing SEM. Normalised using *GAPDH*. Veh = Vehicle control (0.05% DMSO), R1881 = 1 nm RI881 treatment, and siRNA knockdown for 72 h.

A previous study by Lee and coauthors showed KDM7A knockdown led to loss of AR transactivation of target genes, therefore suggesting a role for KDM7A as an AR coregulator in androgen dependent LNCaP cells [31]. To confirm these findings, we knocked down KDM7A and KDM1A, which is a known AR coregulator, and examined the effects of individual and dual knockdown at the protein level (Fig. S8A–D) for KDM7A and KDM1A respectively in LNCaP and C4‐2 and mRNA expression for *KDM7A* (Fig. S8E–G) and *KDM1A* (Fig. S8H–J). We confirmed that siRNA knockdown of KDM7A in LNCaP led to a decrease in *KLK3/PSA* (Fig. 4A). Attenuation of R1881 induced levels of *KLK3/PSA* were also observed with knockdown of KDM1A and the dual knockdown in LNCaP cells (Fig. 4B,C). Interestingly, in the AR‐positive CRPC C4‐2 cell line, *KLK3/PSA* levels were also attenuated, but more subtly with KDM7A knockdown, and an increase of these levels was seen with knockdown of KDM1A (Fig. 4D,E), suggesting that KDM1A plays a different role upon AR transcriptome in C4‐2 cells compared to LNCaP cells. In C4‐2 cells, dual knockdown led to a greater attenuation of *KLK3/PSA* (Fig. 4F). R1881‐induced *KLK3/PSA* levels are weakly up‐regulated with knockdown of KDM7A/KDM1A, having little effect on these levels in 22RV1 cells, potentially due to 22RV1 cells expressing ARv7 and other AR variants (Fig. 4G–I). RNA Seq analysis of differential gene expression of R1881 treated cells with scrambled siRNA against R1881 treated cells with dual KDM siRNAs identified 164 annotated genes differentially expressed in LNCaP (79 up‐regulated genes, 87 downregulated genes; Table S1) and 1065 annotated genes differentially expressed in C4‐2 (649 upregulated genes, 416 down‐regulated genes; Table S2). Comparison of annotated genes only gave 18 genes commonly differentially expressed between the cell lines (Log_2_FC > 1, FDR‐*P* value < 0.05; Fig. 4J; Table S2). No significant pathways were identified within the differentially expressed genes. We next examined how the dual knockdown affected alternative splicing. Interestingly, we saw a greater number of genes differentially alternatively spliced; in LNCaP, 956 significant events were identified for 852 genes and C4‐2 showed 2254 significant events in 1696 genes, with the major alteration being skipped exons (FDR *P* < 0.05, abs value > 0.05; Tables S3– S7 and S8– S12 respectively). These findings confirm KDM7A as an AR co‐regulator in the androgen dependent cell line, LNCaP, but also show that in the androgen independent C4‐2, KDM7A has a greater effect on a subset of AR target genes when KDM1A is also down‐regulated in CRPC.

### 3.3 Pharmacological inhibition of KDM7A and KDM1A leads to loss of proliferation and invasion in AR‐positive CRPC cell lines

**Fig. 5:**
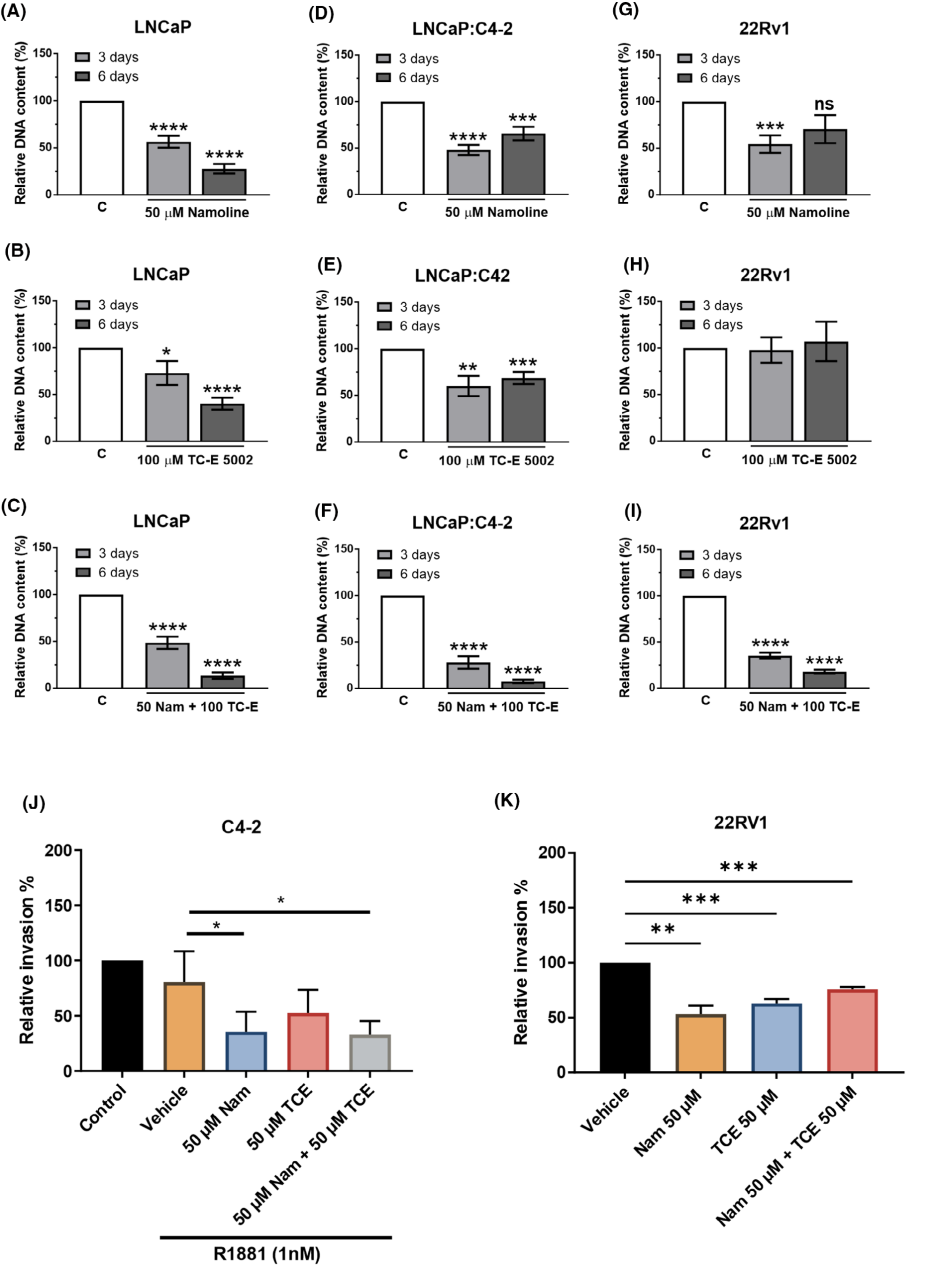
Phenotypic effects of namoline and TCE‐5002 individual and combination treatment on cell proliferation and invasion. Cell proliferation was assessed using CyQUANT assay. Cell lines were treated with namoline, TCE‐5002 and both inhibitors in combination for LNCaP (A–C; *n* = 12); LNCaP:C4‐2 (D–F, *n* = 9) and 22RV1 (G–I, *n* = 9) respectively. In all cell lines LNCaP (C), LNCaP:C4‐2 (F) and 22Rv1 (I) proliferation was 80–90% reduced (*P* ≤ 0.05) by combining Namoline + TC‐E 5002. Invasion assay performed for 24 h with inhibitor treatment in CRPC cell lines (*n* = 3 biological replicas). Five regions of each transwell were counted and analysis only performed if controls contained greater than 100 cells. In C42 and 22RV1 Namoline and TC‐E 5002 showed loss of invasion individually, but no further loss seen in combination (J, K; Nam = Namoline (KDM1A‐selective inhibitor), TC‐E = TC‐E 5002 (KDM7A‐selective inhibitor), Vehicle control (0.05% DMSO). *P ≤ 0.05, **P ≤ 0.005, ***P ≤ 0.001, ****P ≤ 0.0001, ns = not significant, by paired *t*‐test for cyquant. Invasion assay, ANOVA with Bonferroni's *post hoc* test and analysis identified significant differences in treatments. Error bars are SEM.

We next investigated the inhibition of KDM7A and KDM1A utilising pharmacological inhibitors. TCE‐5002 is a selective inhibitor of KDM7A demethylase function, but also targets KDM7B/PHF8 and KDM2A, both of which have previously been shown to be involved in PCa [10, 47, 48, 49, 50], at concentrations < 50 μm (IC50 0.2, 1.2 and 6.8 μm, for KDM7A, KDM7B and KDM2A respectively, IG50 HeLa 40 μm; [51]). Namoline is a KDM1A selective inhibitor shown to affect KDM1A demethylase function and therefore AR co‐regulation and AR‐driven proliferation [52]. Namoline was used for KDM1A inhibition. We first measured the effects of cell proliferation at 3 and 6 days of inhibitor treatment with concentrations that gave significant growth inhibition of 50% for the LNCaP (*P* < 0.05; Fig. 5A,B). In LNCaP combination treatment of namoline and TCE‐5002 led to significant loss of proliferation, with greater loss seen by day 6 (Fig. 5C). For C4‐2 cells, individual treatment led to loss of proliferation (Fig. 5D,E), with greater loss also seen with combination treatment (Fig. 5F). For 22RV1, loss of proliferation was seen with namoline treatment at 3 days but not with TCE‐5002 (Fig. 5G,H), suggesting KDM1A inhibition as an AR co‐regulator could be compensated by AR variants with prolonged treatment as seen by day 6 (Fig. 5G). Combination treatment also led to greater loss of proliferation in 22RV1 (Fig. 5I). Due to significant loss of proliferation with combinatory treatments, we utilised a lower concentration of TCE‐5002 moving forward for the invasion assay. We performed transwell invasion assays on the CRPC cell lines, C4‐2 and 22RV1, with individual and combination treatment of namoline and TCE‐5002 for 24 h. Loss of invasive potential was seen within 24 h in namoline and TCE‐5002 individual treatments, with combination treatment not attenuating the invasive potential further (Fig. 5J,K). These findings suggest that namoline and TCE‐5002 have a greater effect on proliferation when CRPC cells are treated in combination, with KDM inhibition also leading to loss of invasiveness of the CRPC cells.

### 3.4 Pharmacological inhibition of KDMs with combination namoline and TCE‐5002 treatment leads to loss of AR and de‐regulation of AR transcriptome

**Fig. 6:**
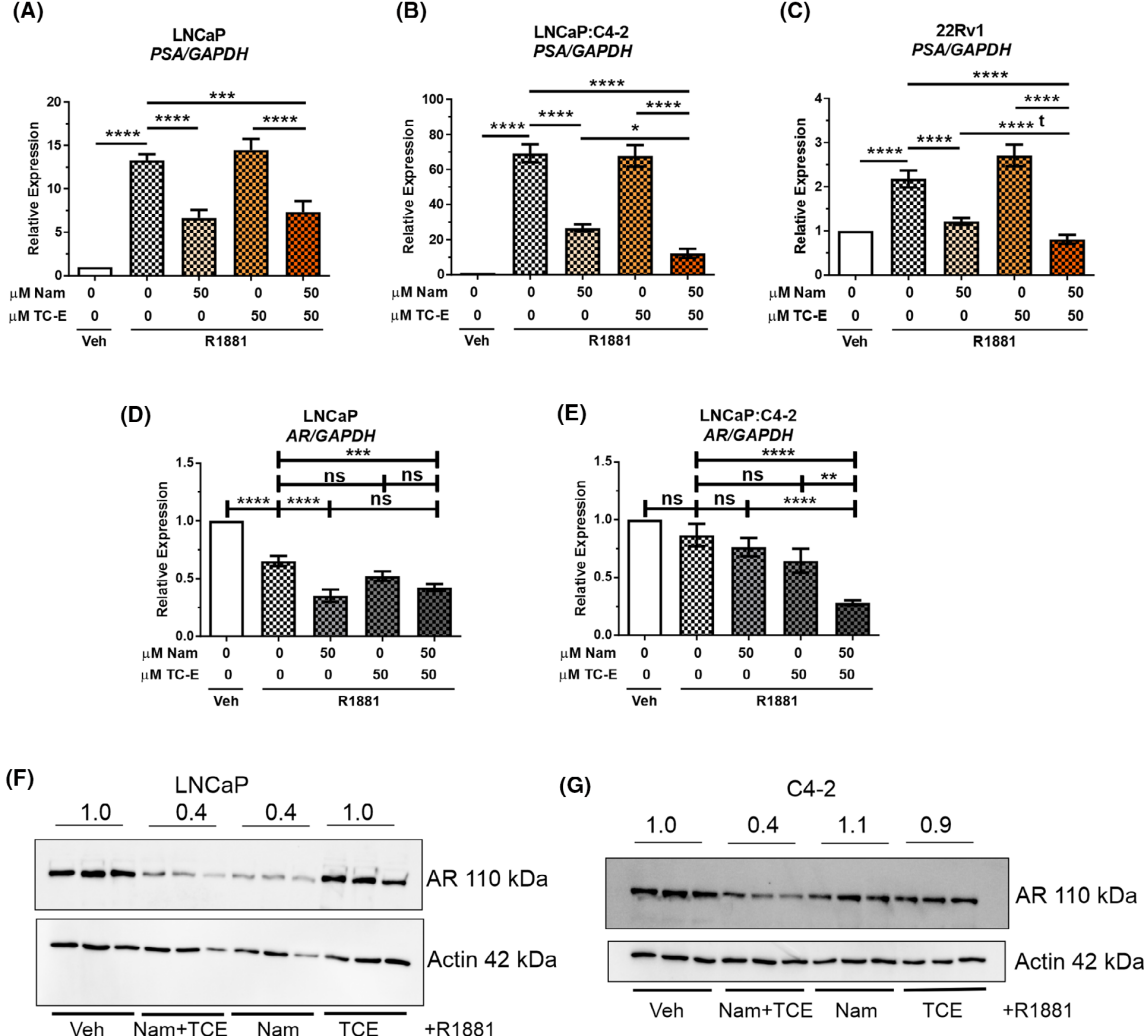
Effect of namoline and TCE‐5002 combination treatment on AR and AR target genes. Effect of combined KDM1A‐and KDM7A‐selective inhibitors on *KLK3/PSA* and *AR* in LNCaP, LNCaP:C4‐2 and 22Rv1. Gene expression changes upon inhibitor and (R1881, 1 nm) treatment were examined using QRT‐PCR (*N* = 9 LNCaP, *n* = 6 LNCaP:C4‐2 and 22RV1, all biological replicas). Whilst in LNCaP (A)combining Namoline with TC‐E 5002 had the same effect on R1881‐induced *PSA* expression as Namoline on its own, both in LNCaP:C4‐2 (B) and 22Rv1 (C) an enhanced inhibitory effect was achieved by the combination, (D) *AR* down‐regulated with namoline only in LNCaP and (E) in combination for C42. Normalised to GAPDH; *P ≤ 0.05, **P ≤ 0.005, ***P ≤ 0.001, ****P ≤ 0.0001, ns = not significant, by ANOVA with Bonferroni's *post hoc* test and paired *t*‐test (*t*‐tests are denotated by t). All error bars show SEM. Western blot for AR protein levels shows lower levels in namoline treated LNCaP cells (F) and in combination in C4‐2 (G). The numbers above blots show mean signal value for the triplicate (*n* = 3, biological replicas). Normzalised to Actin and relative to Vehicle. Veh = Vehicle; Nam = Namoline (KDM1A inhibitor); TC‐E = TC‐E 5002 (KDM7A selective inhibitor).

As AR also plays a role in AR‐positive CRPC, we continued to investigate whether AR‐target gene expression was altered upon inhibition of the KDM coregulators and lead to the loss of the aggressive cell phenotype. We first measured the expression of a known AR‐target gene, *KLK3/PSA*, in LNCaP, C4‐2 and 22RV1 cells treated with a combination of namoline and TCE‐5002 upon androgen induction compared to treatment with each drug alone. Androgen dependent LNCaP cells showed attenuation of *KLK3/PSA* expression with namoline only, whereas *KLK3/PSA* expression was attenuated to a greater extent in C4‐2 and 22RV1 with combination treatment, compared to namoline only (Fig. 6A–C, respectively). To further investigate the effects on AR‐target genes, we followed up these findings in LNCaP and its CRPC derivative C4‐2 for comparative analysis. AR target genes *TMPRSS2*, *FOXA1* and *NKX3.1* showed differential expression patterns with the individual and combination KDM inhibitor treatments (Fig. S9). *TMPRSS2* androgen induction was attenuated only with KDM1A inhibition with namoline in both cell lines (Fig. S9A,B), whereas *FOXA1* also showed attenuation of expression with TCE‐5002 in C4‐2 (Fig. S9C,D). *NKX3.1* showed similar patterns of attenuation in the LNCaP cells, but in C4‐2 *NKX3.1* expression was reduced with both namoline and TCE‐5002, with greater loss in combination treatment (Fig. S9E,F). As shown by other studies, KDM1A is an important AR coregulator, and our data support this for LNCaP; but in CRPC cells, potential alterations in the coregulatory roles of the KDMs show that KDM7A is also involved in co‐activation of a subset of AR‐target genes. To investigate whether the AR‐target genes were reduced with the inhibition of KDM AR coregulators upon treatment, we next measured AR mRNA and protein levels in the treated LNCaP and C4‐2 cells. As previously shown, KDM1A inhibition in LNCaP showed a downregulation of *AR*. However, in C4‐2, *AR* downregulation was only seen in the combination of namoline and TCE‐5002 treatment (Fig. 6D,E respectively). Additionally, this was observed at the protein level, with AR levels reduced with namoline treatment in LNCaP (Fig. 6F) but only in combination treatment in C4‐2 cells (Fig. 6G).

**Fig. 7:**
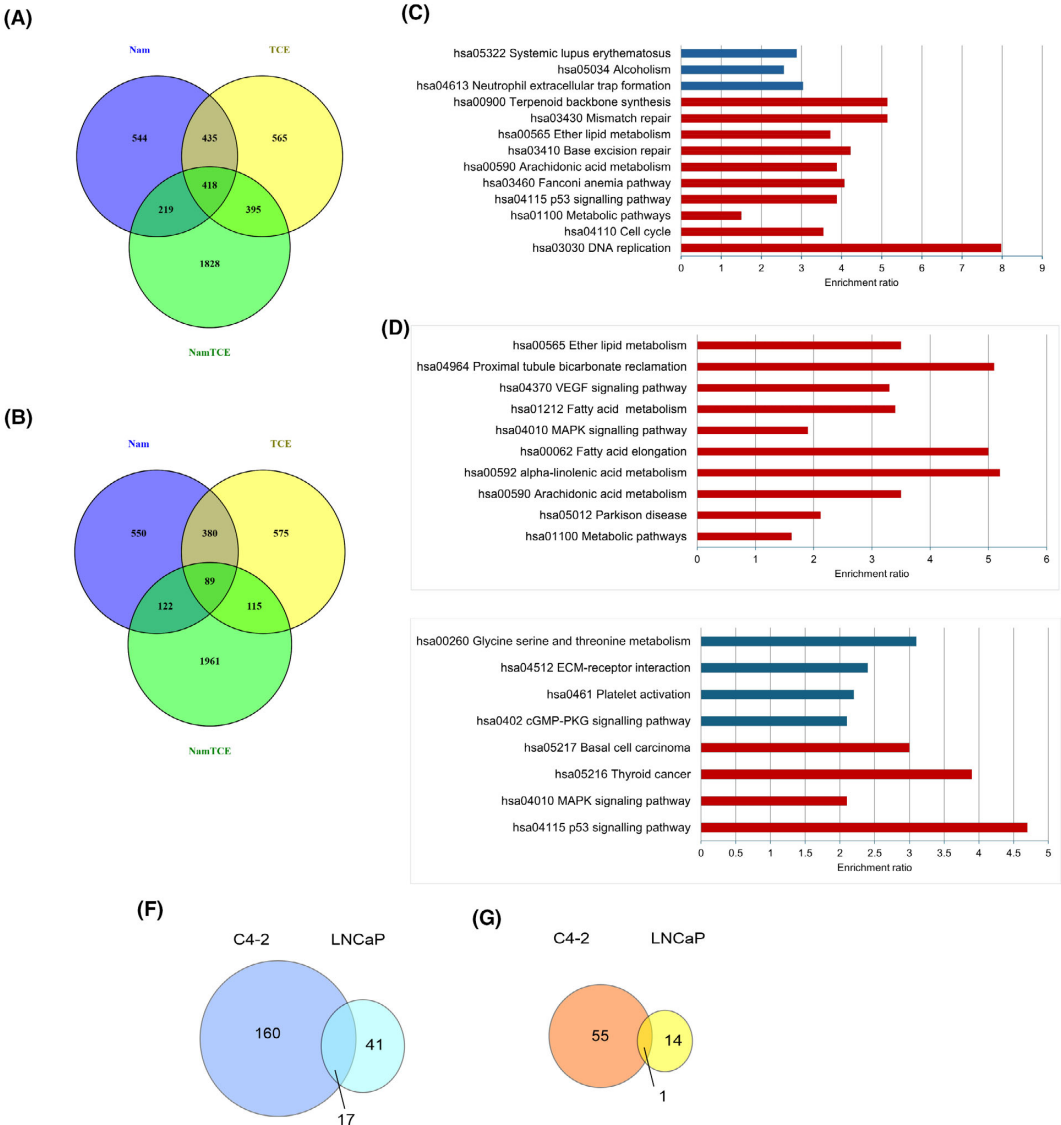
Effect of combined KDM1A‐and KDM7A‐selective inhibitors on CRPC transcriptome and AR target genes. RNA seq analysis identified differential changes to the PCa transcriptome with comparative analysis performed using Venn diagram for (A) down‐regulated and (B) up‐regulated genes with inhibitor treatment (log_2_FC ≥ 1, FDR‐*P* < 0.05). KEGG pathways that were significantly (FDR *P* < 0.05) enriched in overrepresentation analysis of (C) namoline treatment; (D) TCE‐5002 treatment; (E) nam_TCE combination treatment (red bars represent pathways for genes up‐regulated and blue bars for genes down‐regulated). In CRPC, 1371 genes contain ARE sites and are differentially expressed (Sharma *et al*. 2013). The differentially expressed genes > 2 fold FDR < 0.05, R1881 inhibitor combination treatment compared to R1881 control. Venn diagrams show differences between LNCaP and C4‐2 (F) down‐regulated genes, (G) upregulated genes. Nam = Namoline (KDM1A‐selective inhibitor), TC‐E = TC‐E 5002 (KDM7A‐selective inhibitor).

To further examine how this has altered the CRPC transcriptome, we utilised RNA‐Seq analysis in C4‐2 for namoline, TCE‐5002 and combination treated cells in the presence of androgen (1 nm R1881), with a comparative analysis completed on combination treatments between LNCaP and C4‐2. For C4‐2, we identified 2757 genes differentially expressed upon namoline treatment (1616 downregulated, 1141 upregulated genes compared to vehicle‐treated cells; log_2_FC ≥ 1, FDR‐corrected *P*‐value < 0.05; Table S13.1). A similar number of genes was also altered with TCE‐5002 treatment, 2973 genes (1814 downregulated, 1159 upregulated; Table S13.2). For the combination treatment, we saw a greater number of differentially expressed genes, 5147 (2860 downregulated, 2287 upregulated; Table S13.3). We next compared the differential genes (annotated genes) among the three treatment groups to identify subset of genes that are namoline or TCE‐5002 specific. We also determined whether the combination treatment alters a different subset of genes (Table S14). The comparison showed that there were differential genes that were due to the individual treatments and that two‐thirds of the genes in the combination treatment gave a unique differential signature (Fig. 7A,B; Table S14). To identify which pathways could be affected by this transcriptome wide changes, we performed overrepresentation pathway analysis on the up and down regulated genes (FDR‐corrected *P* < 0.05, assigned as significant pathway). The pathway analysis showed that namoline treatment up‐regulated genes involved DNA replication, cell cycle, p53 signalling pathway and metabolism (Fig. 7C). For TCE‐5002 differential genes the pathways were only significant for upregulated genes, with MAPK pathway, VEGF signalling pathway and metabolic pathways identified (Fig. 7D). For the combination treatment, the differential genes were also involved in MAPK signalling and p53 signalling pathways for upregulated genes, as seen for individual treatments but the genes within the pathway are different, with genes that promote apoptosis up‐regulated in the combination treatment (Table S13.3 and Fig. 7E).

We next performed a comparative analysis of combination treatments between LNCaP and C4‐2. We identified 3444 genes differentially expressed in LNCaP combination treatment (2668 downregulated, 1776 upregulated; Table S15). Pathway analysis identified downregulated genes involved in TGF‐β signalling and other pathways in cancer (Fig. S10A). Using Venny 2.0, we conducted a comparison between the differential genes for LNCaP and C4‐2, with the findings showing that only 12% of the genes were common between the two cell lines, and that the differential signature reflects the different pathways being utilised to compensate for loss of KDM function (Fig. S10B,C). As we showed AR loss with the combination treatment in both cell lines, we utilised a list of AR target genes identified for CRPC cells [39], to determine which genes were differentially expressed upon combination treatment. Interestingly, we saw 233 (177 downregulated, 56 upregulated) AR target genes differentially expressed in C4‐2 and 73 (58 downregulated, 15 upregulated) genes in LNCaP. Only 18 of these genes were common to both cell lines (Table S16; Fig. 7F,G). We next examined the effect on alternative splicing of individual and combination treatments. The alternative spliced events examined were alternative 5′ and 3′ splice sites (A5SS and A3SS respectively), skipped exons (SE), retained introns (RI), and mutually exclusive exons (MXE). The number of alternative spliced events are shown in Table 2 for each treatment regimen. C4‐2 individual treatments gave 2212 alternative spliced events with namoline treatment and 2815 events with TCE‐5002 (Tables S17– S21 and S22– S26 respectively). Interestingly, with the combination treatment in C4‐2 the alternative spliced events went up dramatically to 11 988 events, with the majority of the increase in skipped exons (Table 2; Tables S27– S31). Examining these findings, we observed that the combination treatment led to lower levels of exons being skipped (Fig. S11A). Interestingly, this was also shown within the retained intron splicing variants, with combination treatment increasing intron inclusion (Fig. S11B). Closer examination of the altered genes using pathway analysis found significant pathways (FDR *P*‐value < 0.05), in DNA repair (A5SS, hsa03410 Base excision repair; MXE hsa0344 homologous recombination, genes BRCA1, ATM, RAD21); RNA regulation (SE, hsa03013 RNA transport; SE, hsa03018 RNA degradation); Autophagy (SE, hsa03018 Autophagy) and splicing itself (SE, hsa03040 splicesome). This was similar for combination inhibition in LNCaP with 6272 alternative spliced events (Table 2; Tables S32– S36), with pathways identified in splicing (RI, hsa03040 splicesome) and metabolic pathways (hsa01100), showing difference in the altered pathways in comparison with C4‐2.

**Table 2:** The differential alternative splice events for vehicle and combination demethylase inhibitor treatments. Events are alternative 5′ splice site (A5SS), alternative 3′ splice site (A3SS), skipped exon (SE), retained intron (RI), mutually exclusive exon (MXE). Differential analysis was performed between vehicle treated and both individual and combination Namoline (NAM) and TC‐E 5002 (TCE) treatments.

Alternative splice event	C42 NAM	C42 TCE	C42 NAM_TCE	LNCaP NAM_TCE
A5SS	226	306	1052	711
A3SS	178	241	1031	644
SE	1377	1781	7842	3730
RI	204	226	919	620
MXE	227	261	1144	567
Total events	2212	2815	11 988	6272
Total genes	1703	2135	6854	4442

These findings show that namoline and TCE‐5002 combination treatment not only alters the AR transcriptome but in the CRPC cell line, the combination treatment also alters AR‐independent pathways such as MAPK and p53 signalling pathways.

## 4 Discussion

Targeting androgen production and AR to disrupt the AR‐signalling pathway remains the primary treatment approach for PCa. However, this strategy inevitably leads to the development of treatment resistant tumours, CRPC and NEPC [3]. Identifying new therapeutic options for advanced PCa is therefore crucial. We and others have demonstrated that targeting AR coregulators offers an alternative route to disrupt AR‐signalling [18, 21, 31, 38, 53, 54]. Here, we show that genetic alterations of *KDM7A* and *KDM1A* were different between primary and NEPC, with *KDM7A* amplifications in a subset of prostate adenocarcinomas to low *KDM7A* mRNA being the only alteration identified in NEPC. These findings suggest that KDM7A plays a role in PCa and CRPC, but where its loss of expression may be required in NEPC. This could be potentially explained by indirect regulation of *REST* by KDM7A [55]. This study builds on previous findings by examining KDM7A and KDM1A protein levels in larger PCa cohorts from UK and USA, confirming higher levels of KDM7A and KDM1A within PCa [15, 16, 31]. KDM7A has previously been identified as an AR coregulator [31], we then further confirm KDM7A as an AR co‐activator in CRPC cell models and demonstrated that *KDM7A* is regulated by androgen in AR‐positive, androgen‐dependent LNCaP cells. In contrast, KDM1A, which is known to regulate AR [56], no longer regulates AR in the CRPC C4‐2 cell line. This suggests that in androgen‐independent, AR‐positive CRPC cell model, the regulation of AR has been altered. Previous findings by our group showed that pharmacological inhibition of KDM5 and KDM1A in combination reduced AR levels within C4‐2 cells [38]. Consistent with these findings, this study demonstrated that combined pharmacological inhibition of KDM7A and KDM1A resulted in reduction in AR, differential expression of AR‐target genes and a decrease in cancer‐promoting cell phenotype. Utilising the CRPC C4‐2 cell model, we went on to investigate the differences in the transcriptome upon targeting KDM7A and KDM1A individually and in combination treatment. Namoline treatment of C4‐2 cells led to up‐regulation of genes involved in p53 signalling and cell cycle. C4‐2 has functional p53, with p53 known to be a non‐histone target for KDM1A [57]. This is also true in PCa [58], where LSD1 non‐demethylase function also regulates p53 [59]. Notably, with TCE‐5002 treatment, the MAPK signalling pathway was significantly altered, a pathway known to be up‐regulated in CRPC [60]. In the combination treatment of LNCaP and C4‐2 cell lines, we observed greater disruption of AR transcriptome in the CRPC C4‐2 cells, showing that KDM7A was also required by the cells for AR regulation. We also saw pathways required for cancer progression downregulated in the combination treatment (pathways reviewed in [61]). Additionally, with combination treatment we found an increase in alternative splice sites, especially an increase in skipped exons, where genes that were alternatively spliced were in significantly altered pathways (FDR‐*P* value < 0.01) involved in RNA transport, degradation and splicesome. Further investigation is still required to understand how changes in histone modification, especially targets of KDM1A and KDM7A (H3K4, H3K9 and H3K27) alter splicing [62].

In this study, we confirm KDM7A as an AR co‐activator and we show that dual targeting of the KDM7 family and KDM1A is a potential combination therapeutic target for disrupting AR‐signalling in androgen‐AR‐driven CRPC.

## 5 Conclusion

Combination therapeutics are paving the way for treating cancers where vulnerabilities in advanced cancers are known. One example is the synthetic lethality effect of PARP inhibitors in *BRCA1*‐mutated CRPC tumours. Being able to understand the mechanisms of resistance is crucial to prevent further resistance. With the advent of dual inhibitors, many of which are now partnered with LSD1 [63, 64], identifying the partners during the stages of progression will allow for targeted therapies. This study has shown that combination treatment, inhibiting the KDM7 family and KDM1A, is a potential therapeutic avenue for AR‐positive CRPC.

## Conflict of interest

The authors declare no conflict of interest.

## Author contributions

NPM and LJG were involved in conceptualisation. JNJ, VMM, SB, EMN, CLW, AEH, JL‐R, MST, AN, DMH, CSR, and NPM were involved in data acquisition and curation. JNJ, VMM, CLW, SB, ER, BDR, FK, JLP, CSR, and NPM were involved in formal analysis. NPM, LJG, and DMH were involved in funding acquisition. NPM, CSR, ER, and DMH were involved in supervision. JNJ, VMM, CLW, AEH, SB, CSR, and NPM were involved in writing and editing draft. All authors have read and agreed to the submitted version of the manuscript.

## Supporting information


**Fig. S1.** KDM7A antibody validation in PCa cell lines.

**Fig. S2.** *KDM7A* and *KDM1A* gene alterations in prostate adenocarcinoma, metatastatic and neuroendocrine cohorts.

**Fig. S3.** KDM7A IHC staining *H*‐scores for the Nottingham cohort.

**Fig. S4.** KDM1A IHC staining *H*‐scores for the Nottingham cohort.

**Fig. S5.** KDM7A and KDM1A significant clinical correlations and comparison to AR levels within specimens.

**Fig. S6.** KDM7A and KDM1A IHC staining *H*‐scores for the Weill Cornell cohort.

**Fig. S7.** AR binding site was identified within *KDM7A* gene within intron 1, with the selective ARE motif identified within this region.

**Fig. S8.** KDM7A and KDM1A knockdown confirmation experiments for protein and mRNA levels.

**Fig. S9.** Namoline and TCE‐5002 treatment effects on AR target genes.

**Fig. S10.** Pathway analysis for genes differentially expressed in combination treated LNCaP cell line.

**Fig. S11.** Alternative splicing events of skipped exons and retained introns in C4‐2 inhibitor treated cells.

**Table S1.** Differential gene expression data from RNA‐seq analysis of LNCaP siScrambled veh compared to siScrambled + R1881 (1 nm) and LNCaP siScrambled + R1881 (1 nm) compared to siKDM7A + siKDM1A + R1881 (1 nm).

**Table S2.** Differential gene expression data from RNA‐seq analysis of siScrambled veh compared to siScrambled + R1881 (1 nm) and LNCaP siScrambled + R1881 (1 nm) compared to siKDM7A + siKDM1A + R1881 (1 nm).

**Table S3.** Alternative splicing events data for LNCaP siScrambled + R1881 (1 nm) and LNCaP siScrambled + R1881 (1 nm) compared to siKDM7A + siKDM1A + R1881 (1 nm), for 5′SS (5′SS = 5′ splice site, significant splicing events FDR‐corrected *P* value < 0.05 highlighted in yellow).

**Table S4.** Alternative splicing events data for LNCaP siScrambled + R1881 (1 nm) and LNCaP siScrambled + R1881 (1 nm) compared to siKDM7A + siKDM1A + R1881 (1 nm), for 3′SS (3′ SS = 3′ splice site, significant splicing events FDR‐corrected *P* value < 0.05 highlighted in yellow).

**Table S5.** Alternative splicing events data for LNCaP siScrambled + R1881 (1 nm) and LNCaP siScrambled + R1881 (1 nm) compared to siKDM7A + siKDM1A + R1881 (1 nm), for SE (SE = skipped exon, significant splicing events FDR‐corrected *P* value < 0.05 highlighted in yellow).

**Table S6.** Alternative splicing events data for LNCaP siScrambled + R1881 (1 nm) and LNCaP siScrambled + R1881 (1 nm) compared to siKDM7A + siKDM1A + R1881 (1 nm), for RI (RI = retained intron, significant splicing events FDR‐corrected *P* value < 0.05 highlighted in yellow).

**Table S7.** Alternative splicing events data for LNCaP siScrambled + R1881 (1 nm) and LNCaP siScrambled + R1881 (1 nm) compared to siKDM7A + siKDM1A + R1881 (1 nm), for MXE (MXE = mutually exclusive exon; significant splicing events FDR‐corrected *P* value < 0.05 highlighted in yellow).

**Table S8.** Alternative splicing events data for C4‐2 siScrambled + R1881 (1 nm) and LNCaP siScrambled + R1881 (1 nm) compared to siKDM7A + siKDM1A + R1881 (1 nm), for 5′SS (5′SS = 5′ splice site, significant splicing events FDR‐corrected *P* value < 0.05 highlighted in yellow).

**Table S9.** Alternative splicing events data for C4‐2 siScrambled + R1881 (1 nm) and LNCaP siScrambled + R1881 (1 nm) compared to siKDM7A + siKDM1A + R1881 (1 nm), for 3′SS (3′ SS = 3′ splice site, significant splicing events FDR‐corrected *P* value < 0.05 highlighted in yellow).

**Table S10.** Alternative splicing events data for C4‐2 siScrambled + R1881 (1 nm) and LNCaP siScrambled + R1881 (1 nm) compared to siKDM7A + siKDM1A + R1881 (1 nm), for SE (SE = skipped exon, significant splicing events FDR‐corrected *P* value < 0.05 highlighted in yellow).

**Table S11.** Alternative splicing events data for C4‐2 siScrambled + R1881 (1 nm) and LNCaP siScrambled + R1881 (1 nm) compared to siKDM7A + siKDM1A + R1881 (1 nm), for RI (RI = retained intron, significant splicing events FDR‐corrected *P* value < 0.05, highlighted in yellow).

**Table S12.** Alternative splicing events data for C4‐2 siScrambled + R1881 (1 nm) and LNCaP siScrambled + R1881 (1 nm) compared to siKDM7A + siKDM1A + R1881 (1 nm), for MXE (MXE = mutually exclusive exon, significant splicing events FDR‐corrected *P* value < 0.05, highlighted in yellow).

**Table S13.** Differential gene expression data from RNA‐seq analysis of C4‐2 pharmacological inhibition with KDMinhibitors in the presence of R1881. Table 1 differential gene expression data of vehicle treated compared to namoline treated C4‐2 cells. Table 2 differential gene expression data of vehicle treated compared to TCE‐5002 treated C4‐2 cells. Table 3 differential gene expression data of vehicle treated compared to combination namoline & TCE‐5002 treated C4‐2 cells.

**Table S14.** Comparison of up and down‐regulated between the differentially expressed genes for namoline treated, TCE‐5002 treated and combination treated C42 cells, showing commonly regulated and exclusively regulated genes for each treatment regimen.

**Table S15.** Differential gene expression data from RNA‐seq analysis of LNCaP pharmacological inhibition with KDMinhibitors, in combination, in the presence of R1881.

**Table S16.** Comparison of differentially expressed AR‐target genes between LNCaP and C4‐2 combination treated cells.

**Table S17.** Alternative splicing events data for C4‐2 vehicle compared to namoline treatment in the presence of R1881, for 5′SS (5′SS = 5′ splice site, significant splicing events FDR‐corrected *P* value < 0.05, highlighted in yellow).

**Table S18.** Alternative splicing events data for C4‐2 vehicle compared to namoline treatment in the presence of R1881, for 3′SS (3′ SS = 3′ splice site, significant splicing events FDR‐corrected *P* value < 0.05, highlighted in yellow).

**Table S19.** Alternative splicing events data for C4‐2 vehicle compared to namoline treatment in the presence of R1881, for SE (SE = skipped exon, significant splicing events FDR‐corrected *P* value < 0.05, highlighted in yellow).

**Table S20.** Alternative splicing events data for C4‐2 vehicle compared to namoline treatment in the presence of R1881, for RI (RI = retained intron, significant splicing events FDR‐corrected *p* value < 0.05, highlighted in yellow).

**Table S21.** Alternative splicing events data for C4‐2 vehicle compared to namoline treatment in the presence of R1881, for MXE (MXE = mutually exclusive exon, significant splicing events FDR‐corrected *P* value < 0.05, highlighted in yellow).

**Table S22.** Alternative splicing events data for C4‐2 vehicle compared to TCE‐5002 treatment in the presence of R1881, for 5′SS (5′SS = 5′ splice site, significant splicing events FDR‐corrected *P* value < 0.05, highlighted in yellow).

**Table S23.** Alternative splicing events data for C4‐2 vehicle compared to TCE‐5002 treatment in the presence of R1881, for 3′SS (3′ SS = 3′ splice site, significant splicing events FDR‐corrected *P* value < 0.05, highlighted in yellow).

**Table S24.** Alternative splicing events data for C4‐2 vehicle compared to TCE‐5002 treatment in the presence of R1881, for SE (SE = skipped exon, significant splicing events FDR‐corrected *P* value < 0.05, highlighted in yellow).

**Table S25.** Alternative splicing events data for C4‐2 vehicle compared to TCE‐5002 treatment in the presence of R1881, for RI (RI = retained intron, significant splicing events FDR‐corrected *P* value < 0.05, highlighted in yellow).

**Table S26.** Alternative splicing events data for C4‐2 vehicle compared to TCE‐5002 treatment in the presence of R1881, for MXE (MXE = mutually exclusive exon, significant splicing events FDR‐corrected *P* value < 0.05, highlighted in yellow).

**Table S27.** Alternative splicing events data for C4‐2 vehicle compared to namoline and TCE‐5002 combination treatment in the presence of R1881, for 5′SS (5′SS = 5′ splice site, significant splicing events FDR‐corrected *P* value < 0.05, highlighted in yellow).

**Table S28.** Alternative splicing events data for C4‐2 vehicle compared to namoline and TCE‐5002 combination treatment in the presence of R1881, for 3′SS (3′ SS = 3′ splice site, significant splicing events FDR‐corrected *P* value < 0.05, highlighted in yellow).

**Table S29.** Alternative splicing events data for C4‐2 vehicle compared to namoline and TCE‐5002 combination treatment in the presence of R1881, for SE (SE = skipped exon, significant splicing events FDR‐corrected *P* value < 0.05, highlighted in yellow).

**Table S30.** Alternative splicing events data for C4‐2 vehicle compared to namoline and TCE‐5002 combination treatment in the presence of R1881, for RI (RI = retained intron, significant splicing events FDR‐corrected *P* value < 0.05, highlighted in yellow).

**Table S31.** Alternative splicing events data for C4‐2 vehicle compared to namoline and TCE‐5002 combination treatment in the presence of R1881, for MXE (MXE = mutually exclusive exon, significant splicing events FDR‐corrected *P* value < 0.05, highlighted in yellow).

**Table S32.** Alternative splicing events data for LNCaP vehicle compared to namoline and TCE‐5002 combination treatment in the presence of R1881, for 5′SS (5′SS = 5′ splice site, significant splicing events FDR‐corrected *P* value < 0.05, highlighted in yellow).

**Table S33.** Alternative splicing events data for LNCaP vehicle compared to namoline and TCE‐5002 combination treatment in the presence of R1881, for 3′SS (3′ SS = 3′ splice site, significant splicing events FDR‐corrected *P* value < 0.05, highlighted in yellow).

**Table S34.** Alternative splicing events data for LNCaP vehicle compared to namoline and TCE‐5002 combination treatment in the presence of R1881, for SE (SE = skipped exon, significant splicing events FDR‐corrected *P* value < 0.05, highlighted in yellow).

**Table S35.** Alternative splicing events data for LNCaP vehicle compared to namoline and TCE‐5002 combination treatment in the presence of R1881, for RI (RI = retained intron, significant splicing events FDR‐corrected *P* value < 0.05, highlighted in yellow).

**Table S36.** Alternative splicing events data for LNCaP vehicle compared to namoline and TCE‐5002 combination treatment in the presence of R1881, for MXE (MXE = mutually exclusive exon, significant splicing events FDR‐corrected *P* value < 0.05, highlighted in yellow).

## Data Availability

The datasets presented in this study can be found in the online repository NCBI‐GEO. The GEO accession number for all data presented is GSE194281.
