# Supplementary figures and images for: KDM7A and KDM1A inhibition suppresses tumour promoting pathways in prostate cancer
Source: Mol Oncol. 2026 Mar 23:10.1002/1878-0261.70238. Online ahead of print. doi: 10.1002/1878-0261.70238 (PMC13398633; doi:10.1002/1878-0261.70238)

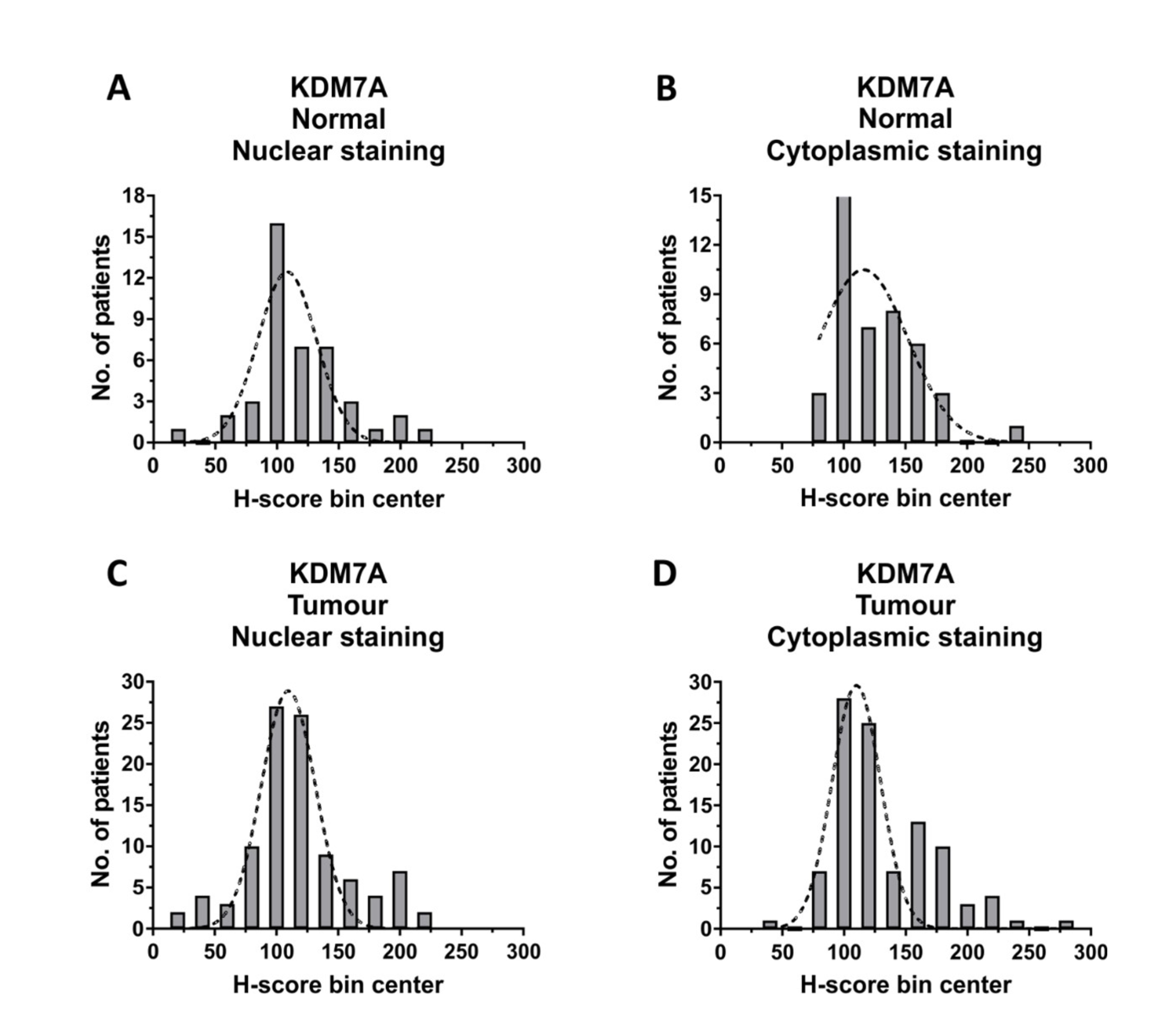

Supplement: Supplementary file 3 — Fig. S3. KDM7A IHC staining H‐scores for the Nottingham cohort. [file MOL2-9999-0-s016.tif]

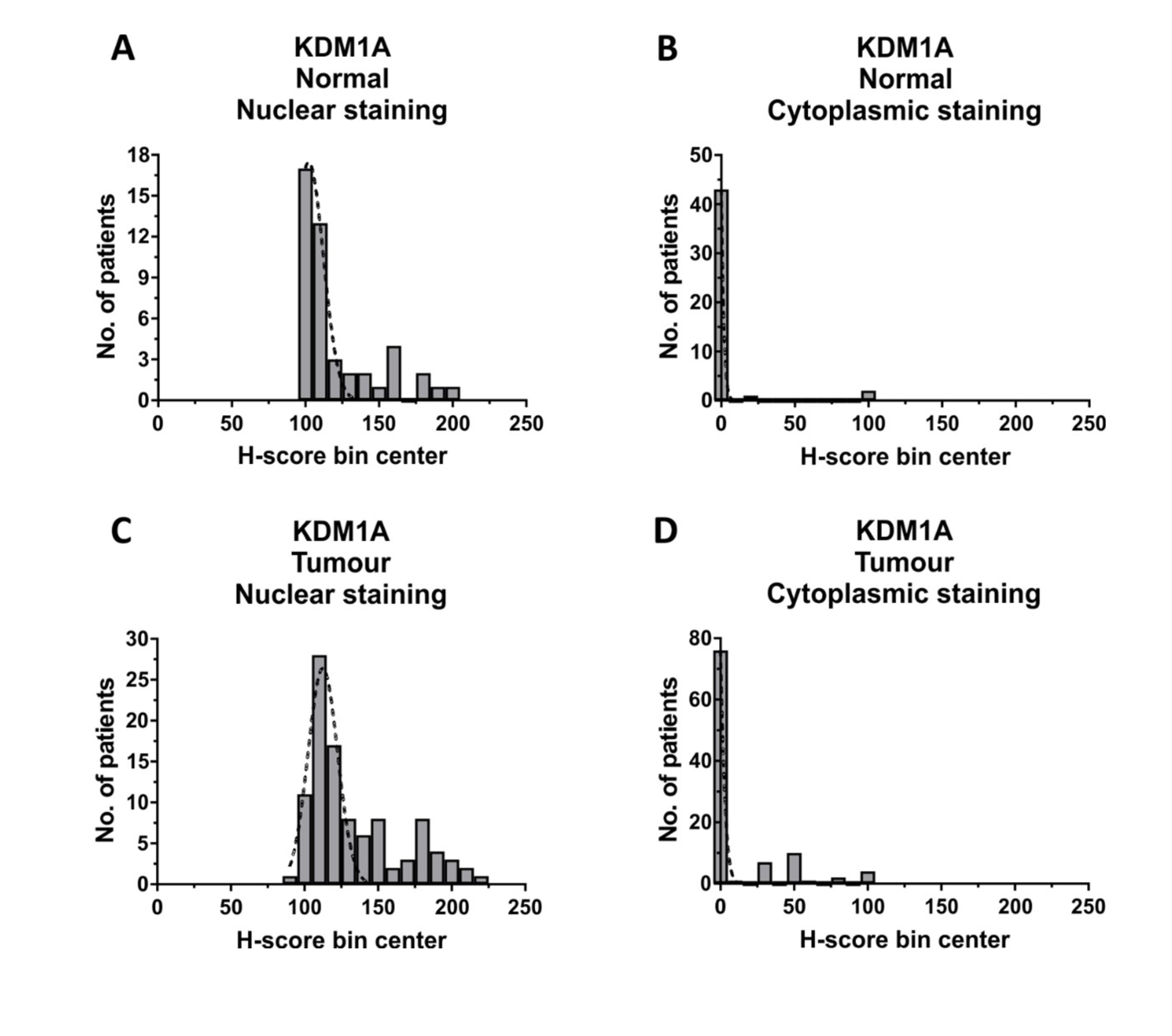

Supplement: Supplementary file 4 — Fig. S4. KDM1A IHC staining H‐scores for the Nottingham cohort. [file MOL2-9999-0-s006.tif]

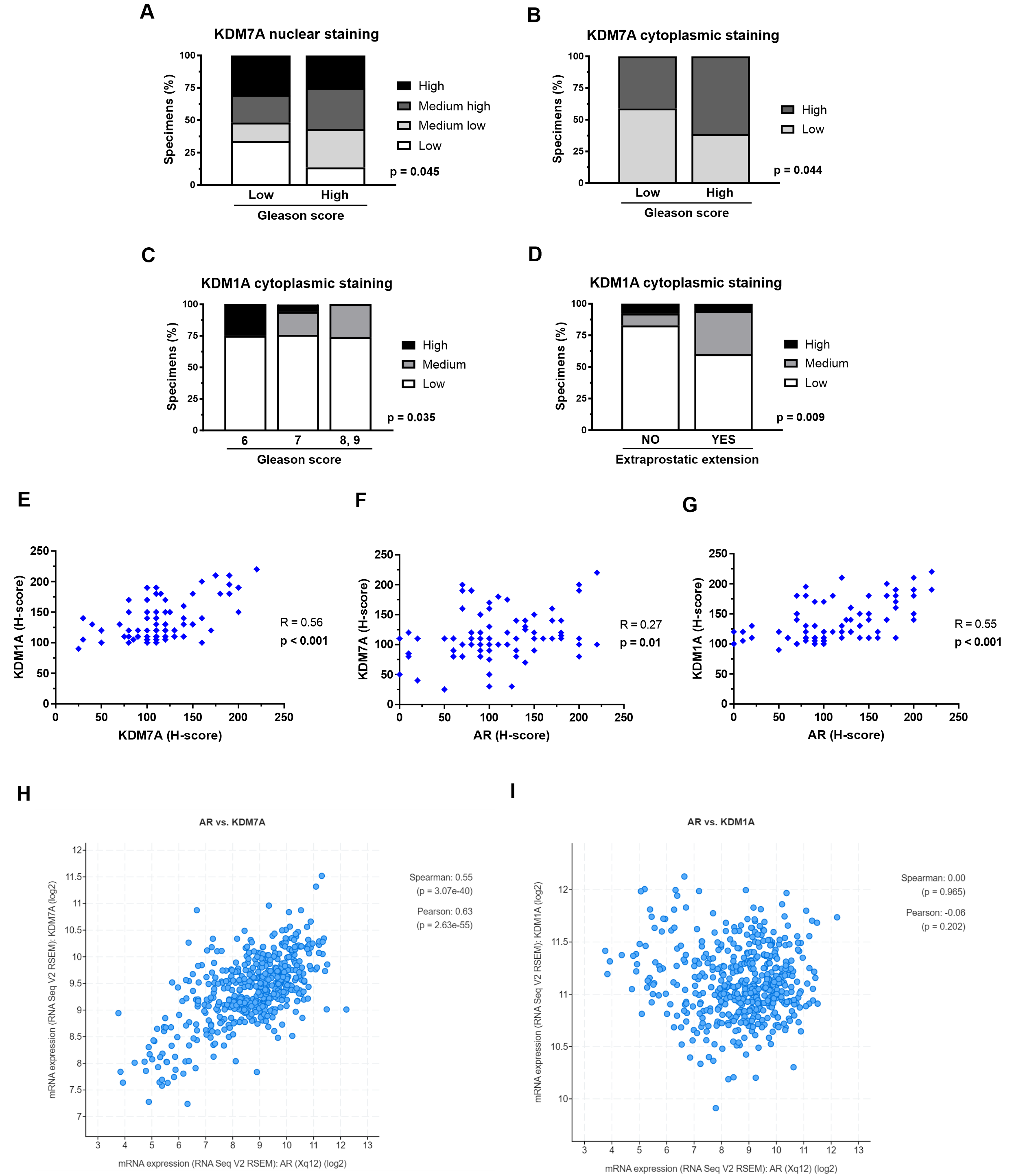

Supplement: Supplementary file 5 — Fig. S5. KDM7A and KDM1A significant clinical correlations and comparison to AR levels within specimens. [file MOL2-9999-0-s036.tif]

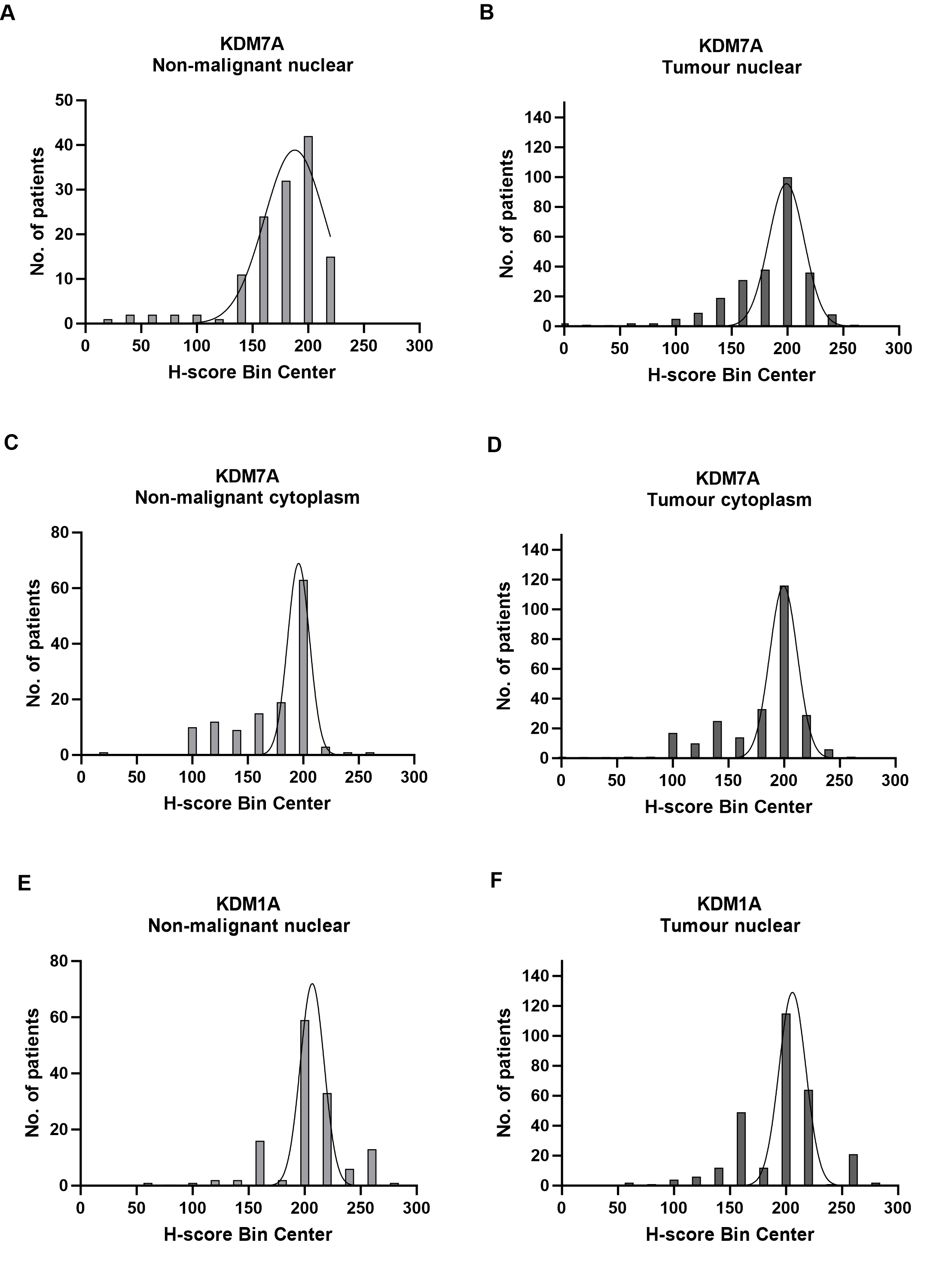

Supplement: Supplementary file 6 — Fig. S6. KDM7A and KDM1A IHC staining H‐scores for the Weill Cornell cohort. [file MOL2-9999-0-s023.tif]

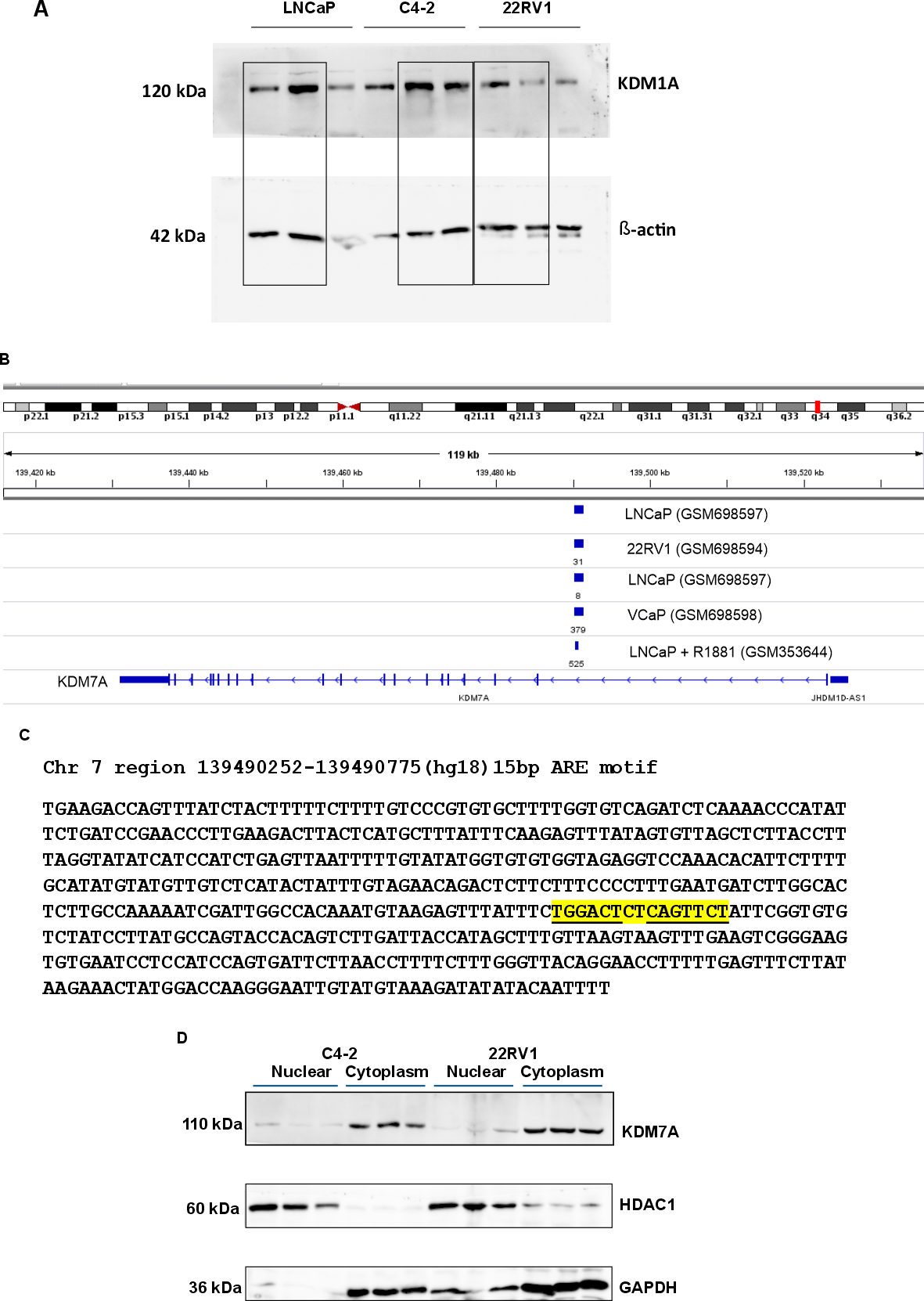

Supplement: Supplementary file 7 — Fig. S7. AR binding site was identified within KDM7A gene within intron 1, with the selective ARE motif identified within this region. [file MOL2-9999-0-s009.tif]

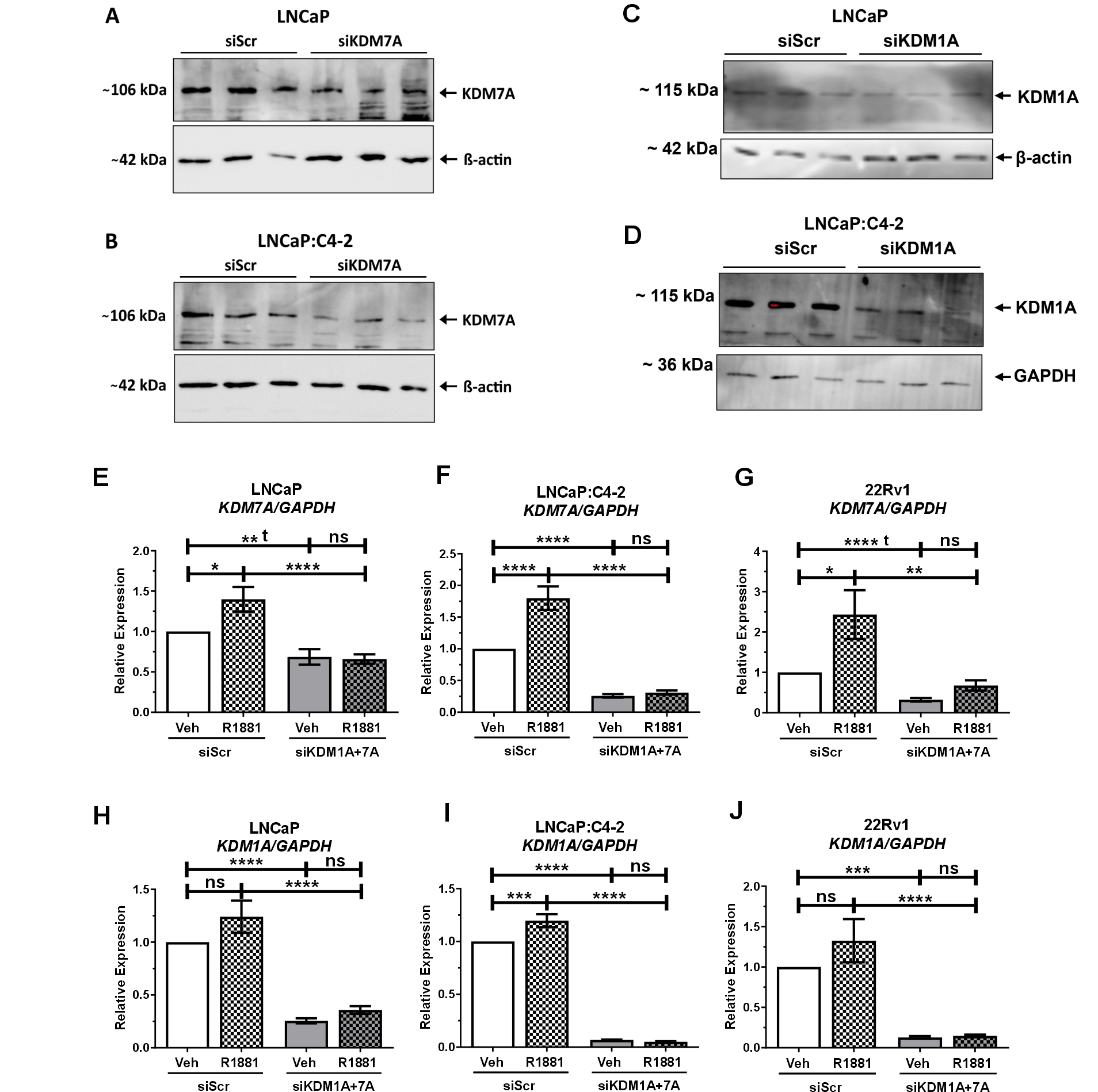

Supplement: Supplementary file 8 — Fig. S8. KDM7A and KDM1A knockdown confirmation experiments for protein and mRNA levels. [file MOL2-9999-0-s020.tif]

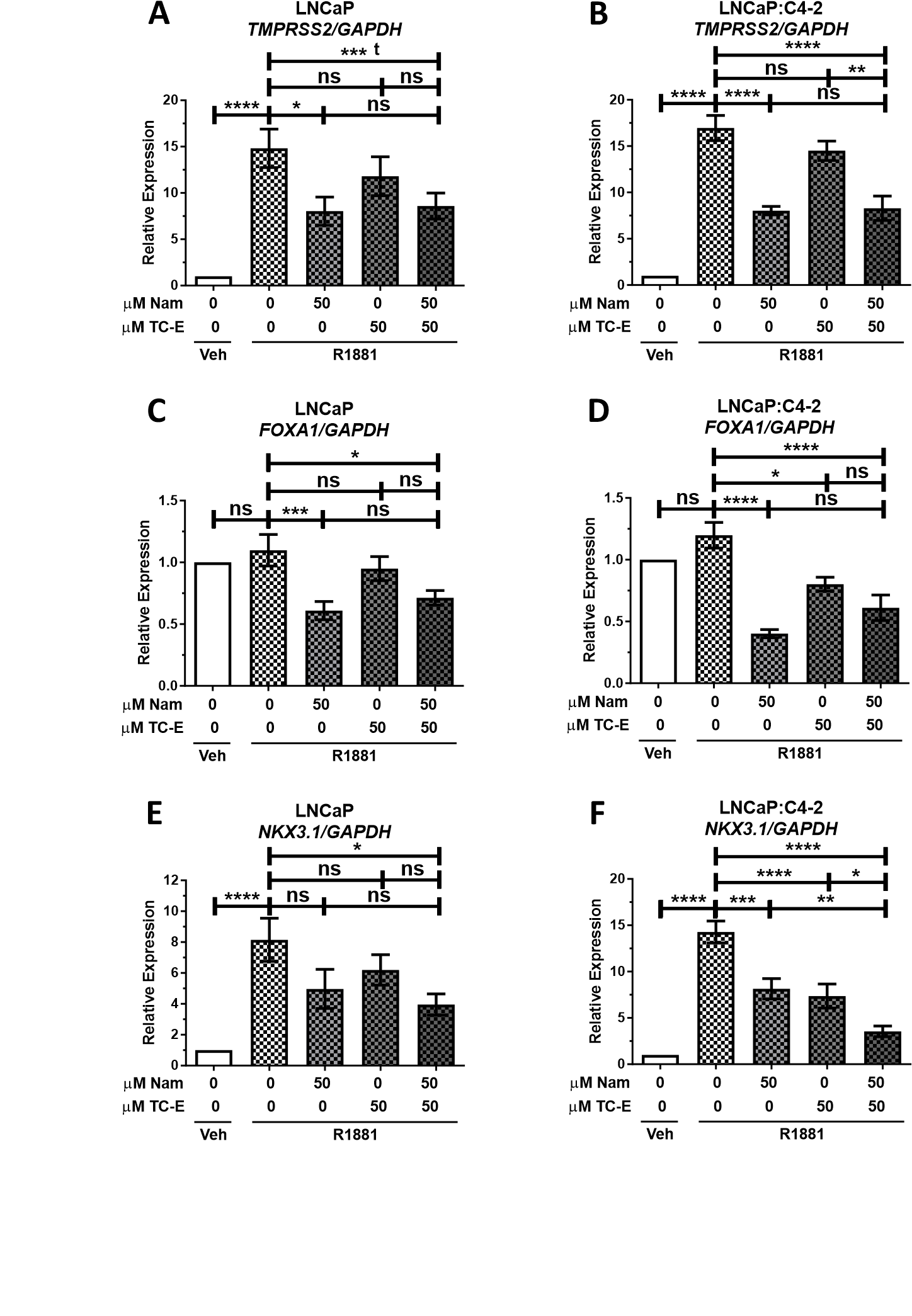

Supplement: Supplementary file 9 — Fig. S9. Namoline and TCE‐5002 treatment effects on AR target genes. [file MOL2-9999-0-s042.tif]

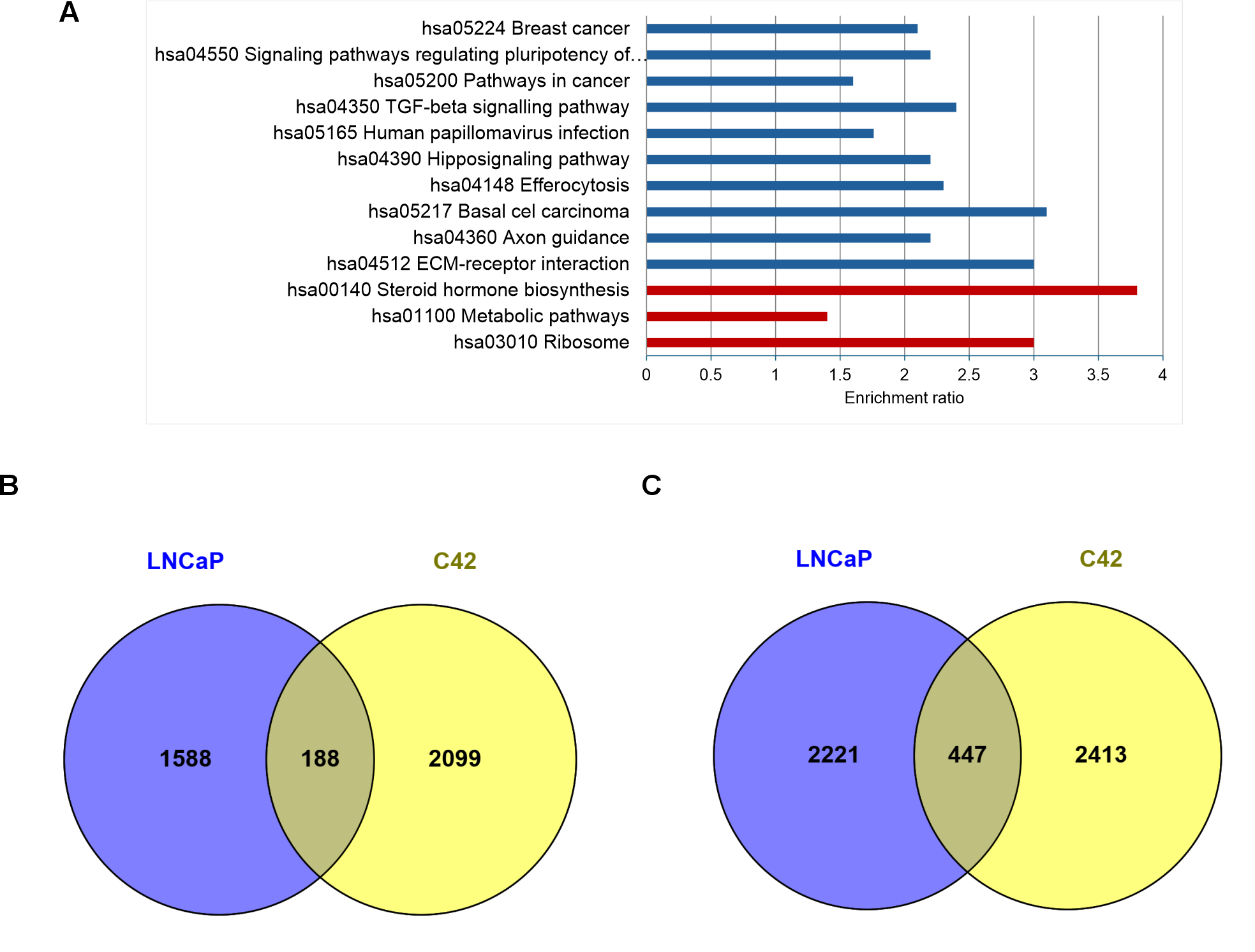

Supplement: Supplementary file 10 — Fig. S10. Pathway analysis for genes differentially expressed in combination treated LNCaP cell line. [file MOL2-9999-0-s028.tif]

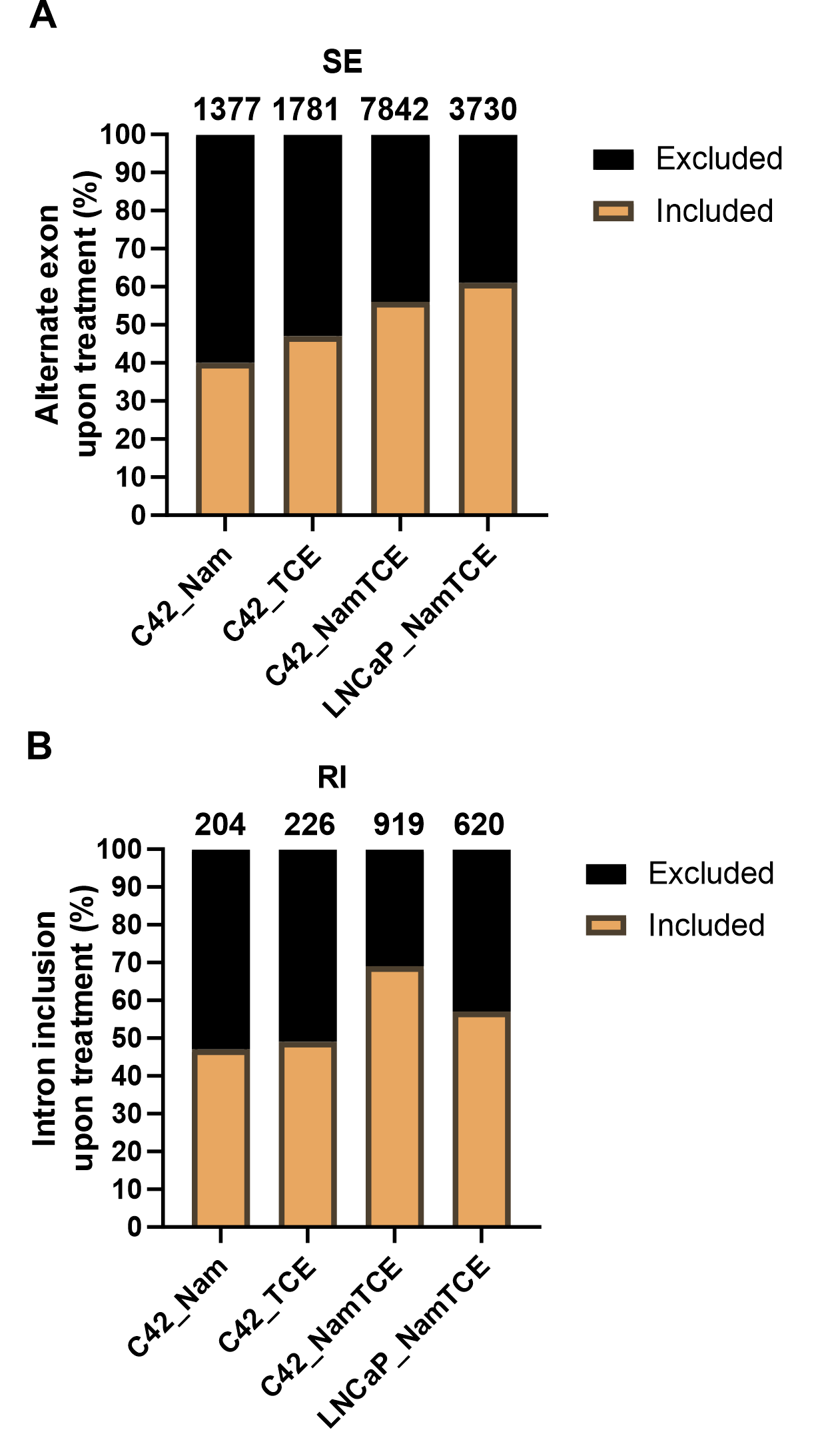

Supplement: Supplementary file 11 — Fig. S11. Alternative splicing events of skipped exons and retained introns in C4‐2 inhibitor treated cells. [file MOL2-9999-0-s008.tif]
